# Synopsis of *Schizanthus* Ruiz & Pav. (Solanaceae), a genus endemic to the southern Andes

**DOI:** 10.3897/phytokeys.154.49615

**Published:** 2020-08-04

**Authors:** Vanezza Morales-Fierro, Mélica Muñoz-Schick, Andrés Moreira-Muñoz

**Affiliations:** 1 Independent researcher. Avenida Vicuña Mackenna Oriente 6640, Santiago, Chile Unafiliated Santiago Chile; 2 Museo Nacional de Historia Natural, Casilla 787, Santiago, Chile Museo Nacional de Historia Natural Santiago Chile; 3 Instituto de Geografía, Pontificia Universidad Católica de Valparaíso, Avenida Brasil 2241, Valparaíso, Chile Pontificia Universidad Católica de Valparaíso Valparaíso Chile

**Keywords:** Argentina, Andes, Chile, classification, endemism, Solanaceae, taxonomy

## Abstract

We present a taxonomic synopsis of the South American genus *Schizanthus* Ruiz & Pav. (Solanaceae), within which we recognise seventeen taxa (14 species with three infraspecific taxa). The genus is mainly distributed in Chile between the coast of the Atacama Desert and the southern temperate forests, while two species occur in the Argentinian Provinces of Mendoza and Neuquén. This taxonomic treatment is based on the analysis of herbarium specimens from 30 different herbaria. For each accepted species we provide details of type specimens and synonymy, key characters, habitat, distribution information and presence in public or private protected areas. We also incorporate a list of representative localities from examined material. We here described three new taxa: Schizanthus porrigens Graham ex Hook. subsp. borealis V.Morales & Muñoz-Schick, **subsp. nov.**, *Schizanthus carlomunozii* V.Morales & Muñoz-Schick, **sp. nov.** and its variety Schizanthus carlomunozii var. dilutimaculatus V.Morales & Muñoz-Schick, **var. nov.**, all of them from the coast of Coquimbo Region. We also recognise Schizanthus litoralis Phil. var. humilis (Lindl.) V.Morales & Muñoz-Schick, **comb. nov.**, as a new combination.

## Introduction

The Chilean flora has been recognised as harbouring a remarkable endemism at all taxonomic levels. This is driven by the long biogeographic history as well as geologically relative recent isolation due to the Andean uplift and associated regional climate changes (Moreira-Muñoz 2011; Scherson et al. 2014). Endemism in the Chilean flora encompasses around 2,000 species, 80 genera and 5 families; and endemic species richness concentrates in Mediterranean Chile between 25°S and 37°S (Moreira-Muñoz 2014). In spite of the Andes acting as a driver of genetic isolation and allopatric speciation, taxa adapted to the harsh high-altitude environment reached the Argentinian eastern side of the Andes, showing a group of plants that can be considered as southern Andean endemics. One of the richest families showing high presence of endemism in the southern Andes is Solanaceae, including rich and subcosmopolitan genera like *Solanum* L. and *Lycium* L., neotropical genera like *Cestrum* L. and *Exodeconus* Raf., Chile-Peruvian genera like species-rich *Nolana* L.f., and southern Andes endemics like *Schizanthus* Ruiz & Pav., *Salpiglossis* Ruiz & Pav. and *Reyesia* Gay (Moreira-Muñoz 2011). Several neotropical groups within the Solanaceae have been recently revised (e.g. Knapp et al. 2019), but some southern Andean endemics such as *Schizanthus* are still lacking an up-to-date revision.

The genus *Schizanthus* was described by Spanish explorers and botanists Hipólito Ruiz and José Antonio Pavón in their Prodromus (Ruiz and Pavón 1794). It is known vernacularly as “*pajarito*” (little bird), “*flor del pajarito*” (little bird flower), “*mariposita*” (little butterfly) and “*orquídea del pobre*” (poorman’s orchid). The first validly published species of the genus was *S. pinnatus* Ruiz & Pav., described in 1798 (Ruiz and Pavón 1798). During the early part of the 19^th^ century, *Schizanthus* species were introduced to Europe for cultivation and several species were later described in Europe from these introductions: *S. porrigens* Graham ex Hook. (Hooker 1824), *S. hookeri* Gillies ex Graham (Graham 1830), *S. grahamii* Gillies ex Hook. (Hooker 1831a) and *S. retusus* Hook. (Hooker 1831b).

It is thought that South America is the ancestral area for all Solanaceae and its major clades (Dupin et al. 2017). Atypical characteristics of the genus *Schizanthus* within Solanaceae, such as bilateral floral symmetry, two fertile stamens and revolute (resupinate) flowers (Grau and Gronbach 1984) have led researchers to believe the genus forms its own monotypic subfamily: Schizanthoideae (Olmstead and Palmer 1992, Hunziker 2001). Molecular studies suggested that *Schizanthus* diverged early from the rest of the Solanaceae (Olmstead and Palmer 1992). The most recent molecular analyses place *Schizanthus* as an early branch and the sister clade of a group including *Duckeodendron* Kuhlm. (Brazil), *Reyesia* (southern Andes endemism) and the Goetzeoideae (a Pantropical group from the Caribbean, Brazil and Madagascar) (Olmstead et al. 2008; Särkinen et al. 2013; Dupin et al. 2017).

The extreme floral diversification in *Schizanthus* is thought to be the product of changes associated with adaptation to different groups of pollinators in Mediterranean and semi-desert habitats of Chile, and high Andean areas of Chile and adjacent Argentina (Pérez et al. 2006). Cocucci (1989) described two pollination syndromes: by bees and by moths. The bee pollination syndrome is the most common and is related to pink-purple corollas, with a nectariferous guide, lower lateral lobes extended as a platform, and explosive discharge of pollen. On the other hand, the moth pollination syndrome is related to white flowers with long tubes, lateral lobes of the upper lip very divided and reflexed, the lower lip reduced, tube curved downwards, without a nectariferous guide, but depending on the moth touch in the lower lateral lobes. Pérez et al. (2006) made field observations of a third pollination syndrome, associated with hummingbirds related to *S. grahamii* and to a lesser extent to *S. hookeri*. High population differentiation is prevalent in populations of *S. grahamii* (Pérez 2011). According to Pérez et al. (2006), a few species with white corollas do not show, in the field, any apparent relation to pollinator; they propose that the floral morphology of *S. lacteus* and *S. candidus* represents an anachronistic character that is maintained despite the disappearance of the original pollinator. This could be due to the continuous aridification of the Atacama Desert, which would have decreased the presence of pollinators. Тoday these *Schizanthus* species would be dependent on self-pollination for their maintenance.

Currently most *Schizanthus* species occur at the core of Mediterranean Chile, and on the transition towards the Atacama Desert, due to the genus being a main component of the Central Chilean biodiversity hotspot (Moreira-Muñoz 2014). Nevertheless, a specific richness assessment for the genus hasn’t been undertaken yet, and an up-to-date revision is pending. This seems to be crucial for informing biogeography as well as conservation and pollination research (Medel et al. 2018). A preliminary revision was published by Muñoz-Schick and Moreira-Muñoz (2008). The current synopsis is based on the study of all herbarium specimens available in the two main Chilean herbaria, personal collections and herbaria from Argentina, Europe and North America. This synopsis includes 17 taxa, including one new species comprising two varieties, one new subspecies and one new combination.

## Materials and methods

For this taxonomic treatment, 1.612 herbarium records from 30 collections were reviewed (AMD, ASU, BA, BM, CONC, DES, E, F, GH, HAL, IND, K, L, LINN, LP, M, MA, MEL, MEXU, MO, P, S, SGO, SI, TDC, U, US, VT, WAG and the Private collection of Andrés Moreira-Muñoz [AMM]). Collections were examined personally at BA, CONC, LP, SGO, SI and AMM, while the specimens from other herbaria were seen through high resolution images, available via the websites of each herbarium and Global Plants JSTOR (http://plants.jstor.org).

For each taxon, we provide a complete list of synonymy and type specimens but only for accepted names or basionyms. Some of them were difficult to trace because they were described from living material in cultivation. Thanks to the digitisation of herbarium material, we have been able to trace duplicates or original materials. When we were able to check specimens personally or by using high resolution images, these have been marked with an exclamation mark (!). We mentioned the holotypes, lectotypes or neotypes of accepted names, even if these were inadvertent typifications in previous publications (Grau and Gronbach 1984). We have designated new lectotypes where it is necessary. In general, we have lectotypified names with the best preserved, or in some cases the only herbarium sheets we have seen. Where there has been difficulty or where the choice may not be obvious, we explain our reasoning under the taxonomic notes. Type specimens are cited with their barcodes in square brackets and written in an identical way as they appear on the specimen. Sheet numbers are cited together with the barcodes if they exist (e.g. SGO000004532 acc. #143618).

Since the original description of the genus, many species have been incorrectly identified. For this reason, we provide notes about nomenclature and botanical history, after the citation of the type material. Our main aim is to clarify the correct name for each taxon, as most *Schizanthus* species have already been described but confused in many publications. We complement this work by adding a list of illustrations published in classic and widely available journals, giving the correct name for them (Appendix I).

Under key characters, we mention the morphological features that help to differentiate each taxon. Here we put much emphasis on the corolla colour and drawings, because the identification of sterile or fruiting specimens is very difficult due to similar leaf shape and size throughout the species. Measurements given in this section were made from dried herbarium material; other information like colour of the corolla and drawings were taken from herbarium specimens and images taken on fieldwork. The distributions of the recognised taxa were established by studying the localities on the labels from herbarium specimens and georeferenced photos from fieldwork. Using these data, distributional maps were constructed over the platform of the GIS ARCGIS 10.4 (ESRI 2015). Few samples had label coordinates, making it necessary to retrospectively georeference the collections; in these cases, we use the following sources of data: Instituto Geográfico Militar [Chile] (Instituto Geográfico Militar 1983), Diccionario Jeográfico de Chile (Risopatrón 1924) and GeoNames Database (https://www.geonames.org/). The label coordinates were also checked using the aforementioned sources. Habitat information was taken from herbarium specimen labels. As a proxy to conservation status, we list the names of the protected areas, public or private, where the presence of each taxon has been verified by herbarium specimens or photos published online. We have included protected areas cited by other publications although when we could not confirm the information. We cite geographically representative specimens to justify the distributional ranges mentioned on the text. The specimens have been alphabetically arranged by the corresponding country. Within each country the specimens are organized geographically from north to south, mentioning the second and third administrative units level, the last one between square brackets. At the end of the citation of each specimen we provide the herbarium code. The corresponding barcode or accession numbers are available from GBIF (https://doi.org/10.15468/82kgtm), which contains the list of specimens revised.

Names of publications where the species are described, and their authorities follow the International Plant Names Index (https://www.ipni.org/).

For newly described taxa, we provide a short diagnosis of the taxon, type material and a full description; here we cite all specimens examined. In this work, we have described two types of infraspecific taxa when we distinguish morphological characters within a species. When a set of morphological characters combines with a disjunct distribution, we classify these taxa as subspecies. However, when we find morphological variability in the specimens, which is replicated in the same locations, we have treated these taxa as varieties (Beentje 2010).

## Taxonomic treatment

The following description of the genus has been taken from Barboza et al. (2016) and modified according to our observations made from fieldwork and examination of herbarium specimens.

### Schizanthus

Taxon classificationPlantaeSolanalesSolanaceae

Ruiz & Pav., Fl. Peruv. Prodr.: 4. 1794

C528ECFD-0CA0-5965-9098-4043F52EED01

#### Type species.

*Schizanthus pinnatus* Ruiz & Pav.

Annual or biennial herbs, sometimes woody at base, usually sticky, with non-glandular unicellular trichomes and glandular shaggy hairs. Leaves rarely entire or slightly serrate, mostly lobed, pinnatisect to bipinnatifid. Inflorescences terminal, paniculate. Flowers 5-merous; calyx tube almost absent, segments slightly unequal, linear-spathulate or lanceolate; corolla tube shorter to several times longer than the calyx, zygomorphic, papilionate; 10–34 mm long, 10–40 mm wide, the upper lip tripartite, with the middle lobe entire, retuse, bilobed or multilobed, lateral lobes bipartite, sometimes these lobes can be two or more times divided; the lower lip with the middle lobe forming a keel and the lateral lobes linear or spathulate, the latter arched inwards; stamens 4, two superior like staminodes and two fertile inferior, sometimes a third staminode is present; anthers dorsifixed, dehiscing explosively by means of pollinators; gynoecium 2-carpellate, ovary with annular nectary, style filiform, stigma inconspicuous, lacking papillae. Capsule septicidal, 2-valved. Seeds up to 40, ellipsoidal or reniform, compressed.

**Figure 1.**
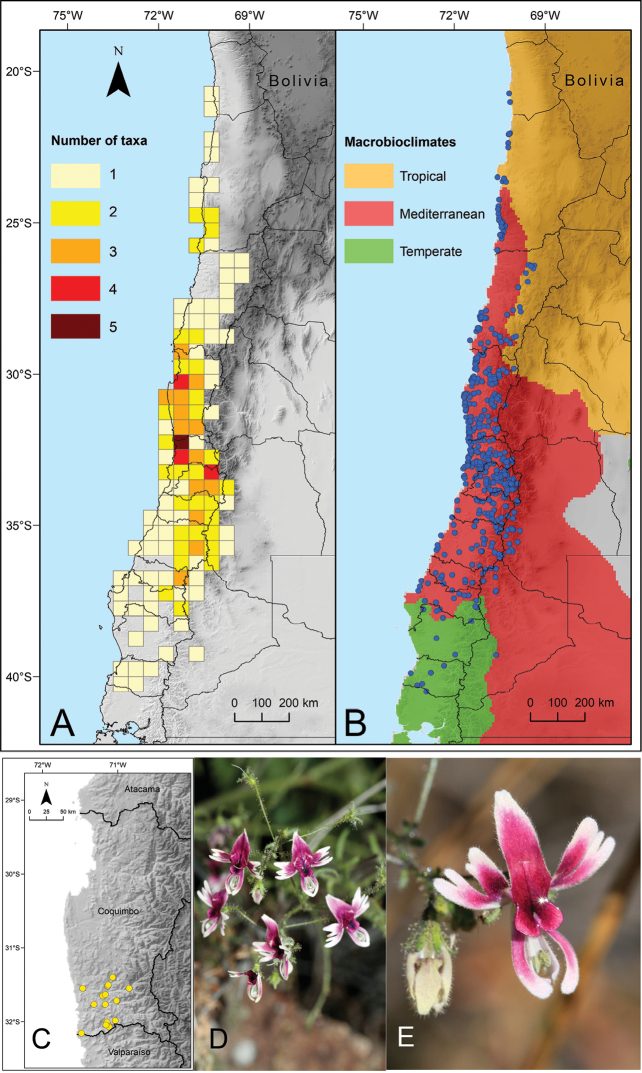
Distribution of the genus *Schizanthus***A** number of taxa of *Schizanthus* by cells of 0.5 degree and administrative units **B** distribution of the genus by macrobioclimates and administrative units **C** distribution of *Schizanthus parvulus***D, E** examples of *S. parvulus* in Las Chinchillas National Reserve. Photos by A. Moreira-Muñoz (**D, E**).

In our treatment we recognise 17 taxa, i.e. 14 species and three infraspecific taxa. The genus is mainly distributed in Chile, where all taxa occur, with three centres of species richness: in the coast of Coquimbo (ca. 30°S), Valparaíso (32–33°S), and Metropolitan Andes (ca. 33°S) (Fig. 1A, Appendix II). In Argentina there are only two species, shared with Chile, which are restricted to the Provinces of Mendoza and Neuquén (Zuloaga et al. 2008). Following the bioclimatic classification by Rivas-Martínez et al. (2011), the genus occurs in three macrobioclimates. The northern portion reaches the Tropical macrobioclimate (Hyperdesertic), while the southern limit of the distribution is extending until the Temperate macrobioclimate (Oceanic). The distribution of the genus (number of records and taxa) is mainly located in the Mediterranean macrobioclimate (Fig. 1B).

### Artificial key to the species of *Schizanthus*

**Table:** 

1	Corolla mostly burgundy, whitish only at the end of each lobe; upper middle lobe prolonged in its base in a distinctive bilobed and bright element; Coquimbo Region (Chile)	**1. *S. parvulus***
–	Corolla not mostly burgundy but other colours; upper middle lobe not prolonged at its base in a different element	**2**
2	Upper middle corolla lobe without a noticeable yellow area	**3**
–	Upper middle corolla lobe with a yellow area that covers 30–95% of its surface, always with segmented veins on it	**9**
3	Upper lip of the corolla about three times longer than the lower lip; upper lateral lobes reflexed	**4**
–	Upper lip of the corolla about the same length as the lower lip, or shorter; upper lateral lobes not reflexed	**7**
4	Corolla bluish to lilac with dark spots or veins (entire or segmented); Antofagasta Region (Chile)	**2. *S. lacteus***
–	Corolla entirely white or with purple spots or veins	**5**
5	Leaf margin deeply divided (pinnatisect), with narrow linear segments; Atacama Region (Chile)	**3. *S. candidus***
–	Leaf margin lobed (pinnatifid)	**6**
6	Corolla tube of equal or similar length to the calyx; Antofagasta Region (Chile)	**2. *S. lacteus***
–	Corolla tube two or three times longer than the calyx; Atacama and Coquimbo Regions (Chile)	**4. *S. integrifolius***
7	Upper middle corolla lobe with the apex deeply bilobed; from Atacama to Valparaíso Region (Chile)	**5. *S. alpestris***
–	Upper middle corolla lobe with the apex entire or slightly notched	**8**
8	Lower lip of the corolla of similar length as the upper lip; upper lateral lobes without spots; Antofagasta Region (Chile)	**6. *S. laetus***
–	Lower lip of the corolla 10–11 mm long, longer than the upper lip; upper lateral lobes with spots, sometimes faint; from Valparaíso to Los Lagos Regions (Chile)	**7. *S. pinnatus***
9	Upper middle corolla lobe rhomboid, apically attenuate and looks about two or three times wider than the lower middle lobe	**10**
–	Upper middle corolla lobe oblong or obovate, not apically attenuate and looks of similar width than the lower middle lobe	**12**
10	Lower middle corolla lobe attenuated into two caudate apex; stamens long and protruding from the corolla tube; from Coquimbo to Biobío Regions (Chile) and Mendoza and Neuquén Provinces (Argentina)	**8. *S. hookeri***
–	Lower middle corolla lobe attenuated into two short pointed apex; stamens short and barely protruding from the corolla tube	**11**
11	Upper middle corolla lobe yellow, except at the base and the apex; this colour not extending to the upper lateral lobes; rarely when the corolla is mostly white; from Metropolitan to Biobío Regions (Chile) and Mendoza and Neuquén Provinces (Argentina)	**9. *S. grahamii***
–	Upper middle corolla lobe completely yellow, this colour extending to the superior part of the upper lateral lobes; Metropolitan Region (Chile)	**10. *S. coccineus***
12	Lower lip of the corolla purple or burgundy, except at the base, markedly darker than the upper lip, which is whitish, pale pink or lilac	**13**
–	Lower lip of the corolla of the same colour as the upper lip	**14**
13	Base of the upper lip of the corolla without or rarely with faint veins, but dotted with dark spots over the yellow area; Valparaíso Region (Chile)	**11a. S. litoralis var. litoralis**
–	Base of the upper lip of the corolla with purple veins, and dotted with dark spots over the yellow area; Valparaíso Region (Chile)	**11b. S. litoralis var. humilis**
14	Upper lateral corolla lobes without spots	**15**
–	Upper lateral corolla lobes with spots, sometimes faint	**16**
15	Corolla pink; peduncles up to 4 mm long; Coquimbo Region (Chile)	**12. *S. splendens***
–	Corolla blue to lilac; peduncles from 4–25 mm long; Coquimbo Region (Chile)	**13b. S. porrigens subsp. borealis**
16	Upper lateral corolla lobes each with a dark medium spot, these not reaching the upper margin	**17**
–	Upper lateral corolla lobes each with a large dark spot, reaching the upper margin	**18**
17	Upper middle corolla lobe with two spots over the margin of the yellow area or a segmented line over it; from Coquimbo to O’Higgins Regions (Chile)	**13a. S. porrigens subsp. porrigens**
–	Upper middle corolla lobe without spots or lines outside the yellow area, but with a white halo over it; Coquimbo Region (Chile)	**13b. S. porrigens subsp. borealis**
18	Upper middle corolla lobe with two lateral spots of dark colour and delimited edges, outside the yellow area; upper lateral lobes with delimited spots; Coquimbo Region (Chile)	**14a. S. carlomunozii var. carlomunozii**
–	Upper middle corolla lobe with a large spot of colour black to burgundy, fading towards the apex, outside the yellow area; upper lateral lobes with fading spots; from Coquimbo to Valparaíso Regions (Chile)	**14b. S. carlomunozii var. dilutimaculatus**

### 1. Schizanthus parvulus

Taxon classificationPlantaeSolanalesSolanaceae

Sudzuki, Agricultura Técnica, Chile 5(1): 33. 1945

4AB19997-0529-5BDD-87BD-42F08DDA16EA

Fig. 1C–E

#### Type.

Chile. Coquimbo: Hacienda Illapel, Caren, frente a El Vato [Bato], ca. 900 m alt., 20–24 Oct 1941, *C. Muñoz & G.T. Johnson 2295* (holotype: SGO! [SGO000004532 acc. #143618]; isotype: SGO! [SGO000004534 acc. #148996]).

#### Taxonomic notes.

In the protologue, the author indicates the holotype as being held at the national herbarium of the National Museum of Natural History, Santiago (SGO). At the same time, she mentions a duplicate at the herbarium of the Departamento de Genética y Fitotecnia del Ministerio de Agricultura. Today, both specimens can be found at SGO. In 2007, Mélica Muñoz identified one of the specimens as the holotype, as it has a label with the name of the species and the descriptor “*S. parvulus* nov. sp. Sudzuki.”, while the second specimen lacks this information.

#### Key characters.

Easily recognisable as having the smallest flower of the genus (10–14 mm long, 10–12 mm wide) and its conspicuous bilobed and brilliant element at the base of the upper middle corolla lobe, that exudes nectar. Corolla mostly burgundy and whitish only at the end of each acute lobe.

#### Distribution.

Endemic to Chile, in the Region of Coquimbo (Province of Choapa, 31°20'–32°10' lat. S). 100–900 m a.s.l.

#### Habitat.

*Schizanthus parvulus* grows in coastal hills and interior valleys of semiarid xerophytic scrub, including small trees of *Quillaja saponaria* Molina (Quillajaceae), shrubs such as *Spinoliva ilicifolia* (Hook. & Arn.) G.Sancho (Asteraceae), *Haplopappus pulchellus* DC. (Asteraceae), *Haplopappus velutinus* J.Rémy (Asteraceae), *Pleocarphus revolutus* D.Don (Asteraceae) and herbs such as *Loasa illapelina* Phil. (Loasaceae), *Calceolaria collina* Phil. (Calceolariaceae) and *Chaetanthera limbata* (D.Don) Less. (Asteraceae).

#### Conservation.

**Chile. Coquimbo**: Las Chinchillas National Reserve, Cerro Santa Inés Natural Sanctuary.

#### Selected specimens examined.

**Chile. Coquimbo: [Choapa Province**] Quebrada El Cobre, Parque Nacional Las Chinchillas, Auco, 31°31'S, 71°6'W, 567 m a.s.l., 1 Oct 2002, *L. Suárez s.n.* (CONC); Illapel, 24 km cruce Carretera Panamericana a Illapel, orillas de línea férrea, 21 Sep 1996, *M. Muñoz 3781* (SGO); Cuesta Guenchigualleco, a 60 km al Oeste de Illapel, ca. 100 m a.s.l., 20–24 Oct 1941, *C. Muñoz & G.T. Johnson 2273* (SGO); Vicinity of Illapel, 7 Oct 1914, *J.N. Rose 19273* (US).

### 2. Schizanthus lacteus

Taxon classificationPlantaeSolanalesSolanaceae

Phil., Fl. Atacam.: 45. 1860

806A3809-5C42-57F7-A498-5C9D33454F89

Fig. 2A–D

Schizanthus sanromanii Phil., Anal. Univ. Chile 91: 126. 1895, as “*san romani*”.

#### Type.

Chile. Antofagasta: In deserto Atacama ad Hueso Parado, Dec 1853, *R.A. Philippi s.n.* (holotype: SGO! [SGO000004524 acc. #055390]).

**Figure 2.**
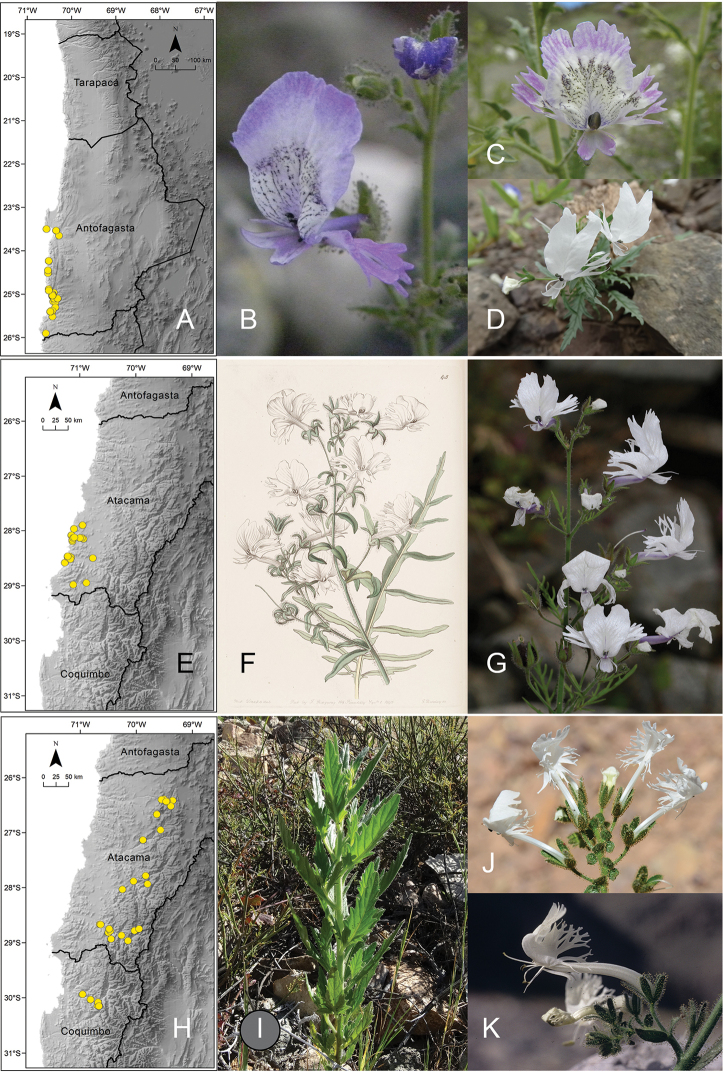
**A** distribution of *Schizanthus lacteus***B** examples of *S. lacteus* in La Chimba National Reserve **C** Quebrada Peralito **D** Paposo **E** distribution of *Schizanthus candidus***F** illustration of *S. candidus* published with the description of the species (Lindley 1843) **G** example of *S. candidus* in Quebrada de Carrizal **H** distribution of *Schizanthus integrifolius***I** examples of *S. integrifolius* in Embalse Santa Juana **J** Río Cachitos **K** Alto del Carmen. Photos by M.T. Eyzaguirre (**B–D, I, J**), A. Moreira-Muñoz (**G**), S. Moreira (**K**).

#### Key characters.

In general, the corolla is entirely white, but it can vary from blue to lilac. Sometimes it has purple spots or veins on the upper lip or is purple throughout the corolla without any drawings. Leaves linear or lanceolate but lobed at margins. Like *S. integrifolius* and *S. candidus*, this species shows a reduced lower lip as compared to the upper lip.

#### Distribution.

Endemic to Chile, in the coast of the Region of Antofagasta (Province of Antofagasta, 23°30'–26°0' lat. S). 20–900 m a.s.l.

#### Habitat.

*Schizanthus lacteus* is part of the lomas vegetation and grows in the fog (camanchaca) zone, on steep hillsides and among rocks alongside watercourses; in gravel soil or coarse sand.

#### Conservation.

**Chile. Antofagasta**: Morro Moreno National Park, La Chimba National Reserve, Paposo Norte Natural Monument.

#### Selected specimens examined.

**Chile. Antofagasta: [Antofagasta Province**] Península Moreno, cerro al O de Juan López, 23°30'S, 70°34'W, 600 m a.s.l., 18 Oct 1992, *G. Baumann 25* (CONC, SGO); El Rincón, just north of Paposo, along trail to old Paranas Mine, 7 Dec 1925, *I.M. Johnston 5480* (F, US); Bajada a Caleta El Cobre, 24°15'S, 70°33'W, 1 Oct 1987, *S. Teillier 492* (CONC, SGO); Cachinal, quebrada frente a la playa, 25°10'S, 70°25'W, 80 m a.s.l., 14 Sep 1994, *L. Loyola 94-12* (CONC); Paposo, entrada a la Qda. Los Peralitos, 25°1'57"S, 70°26'30.3"W, 490 m a.s.l., 30 Sep 2005, *M. Muñoz 4608* (SGO).

### 3. Schizanthus candidus

Taxon classificationPlantaeSolanalesSolanaceae

Lindl., Edwards’s Bot. Reg. 29: tab. 45. 1843

54F104F6-09A3-56AE-9A08-A8510A7D7402

Fig. 2E–G

Schizanthus albiflorus Phil., Anal. Univ. Chile 91: 124. 1895.

#### Type.

Chile. Atacama: Coquimbo?, *T.C. Bridges 1356* (lectotype designated by Grau and Gronbach 1984, pg. 124 [as type]: K! [K00058348, photo at IND! [IND-0107170]]; isolectotypes: BM! [BM000995488, BM000995489], E! [E00089541], G [n.v., F! neg. 23090], P! [P00477035, P00477036]).

**Figure 3.**
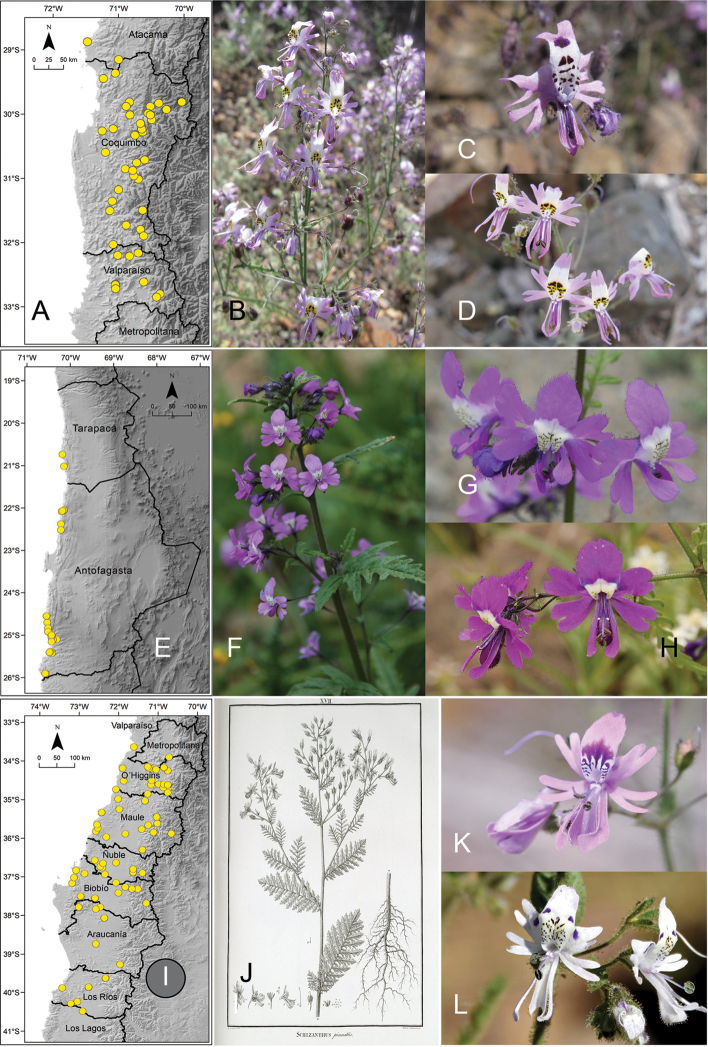
**A** distribution of *Schizanthus alpestris***B** examples of *S. alpestris* in Cuesta Pajonales **C** Cordillera El Melón **D** Las Chinchillas National Reserve **E** distribution of *Schizanthus laetus***F** examples of *S. laetus* in Quebrada Los Yales **G** Quebrada Peralito **H** Taltal **I** distribution of *Schizanthus pinnatus***J** illustration of *S. pinnatus* published with the description of the species (Ruiz and Pavón 1798) **K** examples of *S. pinnatus* on road from Los Angeles to Antuco **L** Cerro Poqui. Photos by A Moreira-Muñoz (**B–D**), M.T. Eyzaguirre (**F, G, L**), M. Aldunate (**H**), S. Moreira (**K**).

#### Taxonomic notes.

There are several specimens collected by Bridges numbered as 1356, but only one of them was labelled with the complete original location (“*Hills near the valley of Huasco Prov. of Coquimbo*”) [E00089541]. The handwriting on the other specimens only states “Coquimbo”, which was (at that time) the name of the Province associated with the locality. In late 1843, the original Province of Coquimbo was divided into two new administrative units, the southern part maintained the name of the original Province (Coquimbo), while the northern section became the Province of Atacama (Pérez-Rosales 1857). Most of these territories are known today as the Regions of Coquimbo and Atacama; the area where the type material was collected corresponds to the latter. This situation can explain why some publications (Grau and Gronbach 1984; Rodríguez et al. 2018) have mentioned *S. candidus* as occurring in the actual Region of Coquimbo, where this species does not grow.

#### Key characters.

This species has a white flower, the corolla tube can be longer or as long as the calyx; the lower lip of the corolla is reduced, compared to the upper part. Pinnatisect leaves with linear lobes.

#### Distribution.

Endemic to Chile, in the coast and interior valleys of the Region of Atacama (Provinces of Copiapó and Huasco, 27°50'–29°0' lat. S). 20–200 m a.s.l.

#### Habitat.

*Schizanthus candidus* grows abundantly among the rocks over fine sand, in dry places or in seasonally wet quebradas with scattered shrubs; it is rarely found along the roads in open areas. It has been found growing with *Leontochir ovallei* Phil. (Alstroemeriaceae), *Cistanthe grandiflora* (Lindl.) Schltdl. (Montiaceae), *Chaetanthera limbata* (D.Don) Less. (Asteraceae) and *Senecio troncosii* Phil. (Asteraceae). Most abundant in rainy years associated to El Niño events, being a main element of the “blooming desert” (Muñoz-Schick 1985; Chávez et al. 2019).

#### Conservation.

**Chile. Atacama**: Llanos de Challe National Park.

#### Selected specimens examined.

**Chile. Atacama: [Copiapó Province**] Coastal road from Carrizal Bajo to Totoralillo, 27°57'56"S, 71°6'10"W, 179 m a.s.l., 27 Nov 2008, *R. Baines*, *M.F. Gardner*, *P. Hechenleitner*, *C. Morter & D. Rae 159* (E); [**Huasco Province**] Carrizal Bajo, Quebrada Oriente, 28°7'17"S, 71°5'57"W, 11 Oct 2002, *A. Moreira 738* (SGO); Quebrada angosta al lado norte de entrada a Aguada Tongoy, entre Huasco y Freirina, 28°30'S, 71°6'W, 90 m a.s.l., 10 Sep 2011, *M. Lazo & C. Stone 64* (CONC).

### 4. Schizanthus integrifolius

Taxon classificationPlantaeSolanalesSolanaceae

Phil., Anal. Univ. Chile 43: 530. 1873

A7688415-CAF0-5B7C-A305-C1B7038530C8

Fig. 2H–K

#### Type.

Chile. Atacama: In andis Copiapo, Dec 1841, *C. Gay 1174 bis* (lectotype here designated: SGO! [SGO000004520 acc. #055382]).

#### Taxonomic notes.

According to the protologue, the species was described using materials from two different collection: Andes de Copiapó by C. Gay (1174 bis) and Quebrada de Puquios by F. Geisse. Grau and Gronbach (1984: 123) selected as lectotype [as type] one of the specimens at SGO. However, they erroneously mixed data from two different specimens. They cited: “*en el interior de la provincia de Atacama*, *quebrada de Puquis*, *F. GEISSE* (*SGO 55382*)”, but the SGO number 55382 corresponds to the specimen collected in the mountains of Copiapó by Gay. On the other hand, specimens from Quebrada de Puquios, gathered by Geisse have the numbers 55383 and 42901. Today, we cannot be sure if they selected the specimen from Puquios and annotated the number erroneously, or if they always wanted to lectotypify the specimen collected by Gay but did not realise it was from a different locality. In our opinion, the lectotypification by Grau and Gronbach (1984) is not valid, as the designated lectotype should be referred to a single collection (Turland et al. 2018; Art. 9.17). For this reason, we have chosen a new lectotype; this herbarium sheet shows the characters of the species more clearly.

The specimens from Quebrada de Puquios [SGO000004521 acc. #055383, SGO000004522 acc. #042901 pro parte] have labels written by R.A. Philippi stating they were collected in 1865, and not in 1861, the year mentioned in the protologue. Most likely, Philippi confused the date of collection in the protologue.

#### Key characters.

This species has a reduced inferior lip, compared to the upper lip and white flowers with a very long corolla tube which is two or three times longer than the calyx. The basal leaves have entire or dentate and undulate margins.

#### Distribution.

Endemic to Chile, in the Andean mountains and valleys from the Regions of Atacama (Province of Chañaral, 26°20' lat. S) to Coquimbo (Province of Elqui, 30°10' lat. S). 800–2800 m a.s.l.

#### Habitat.

*Schizanthus integrifolius* is frequent in rocky areas on hillsides and in screes. It grows better in well-drained soils, like coarse sand, but is less abundant when growing in clay soils. Other species in the community include *Heliotropium sinuatum* (Miers) I.M.Johnst. (Heliotropiaceae), *Encelia canescens* Lam. (Asteraceae) and *Spinoliva ilicifolia* (Hook. & Arn.) G.Sancho (Asteraceae).

#### Conservation.

**Chile. Atacama**: Los Huascoaltinos Private Natural Reserve (Peña-Gómez 2005).

#### Selected specimens examined.

**Chile. Atacama: [Chañaral Province**] El Salvador, 26°23'42.53"S, 69°30'19.33"W, 2145 m a.s.l., 25 Nov 2011, *S. Teillier & A. Walkowiak 8128* (CONC); In Cachiyuyo, im Berraquilla-Tal, 800 m a.s.l., 21 Sep 1972, *O. Zoellner 6122* (L); Potrerillos, camino a la mina El Hueso, 31 Oct 1995, *S. Teillier 3697* (SGO); [**Copiapó Province**] Camino de Copiapó al Tranque Lautaro, 14 km antes del tranque, 26 Oct 1965, *M. Ricardi*, *C. Marticorena & O. Matthei 1494* (CONC); In Jorquera Tal, 1500 m a.s.l., 12 Jan 1970, *O. Zoellner 4258* (L); [**Huasco Province**] San Félix, al interior, 28°5'S, 70°28'W, 1250 m a.s.l., 11 Dec 2008, *M. Rosas 6142* (CONC); Vallenar, Alto del Carmen,, ca. 800 m a.s.l., Nov 1923, *E. Werdermann 151* (E, L, SI); Poco al W de El Tránsito, 3 Oct 1997, *M. Muñoz 3831* (SGO); **Coquimbo: [Elqui Province**] A unos 10 km al sur de Vicuña en el camino a Hurtado, 800 m a.s.l., 13 Oct 1940, *G. Looser 4305* (CONC, SGO); Camino entre Vicuña y Hurtado, Hacienda Pangui, 800 m a.s.l., 13 Oct 1940, *G. Looser 4305* (US).

### 5. Schizanthus alpestris

Taxon classificationPlantaeSolanalesSolanaceae

Poepp., Not. Natur- Heilk. 23: 291. 1829

CB16B057-1C95-5E14-ADB3-90D8F22296D0

Fig. 3A–D

Schizanthus alpestris Poepp. ex Benth., Prodr. [A.P. de Candolle] 10: 202. 1846. nom. illeg. superfl.
Schizanthus alpestris var. glanduliferus Phil., Linnaea 33(2): 214. 1864, as “*glandulifera*”.
Schizanthus angustifolius Phil., Anal. Univ. Chile 91: 119. 1895.
Schizanthus glanduliferus Phil., Anal. Univ. Chile 91: 120. 1895.
Schizanthus laciniosus Phil., Anal. Univ. Chile 91: 125. 1895.

#### Type.

Chile. Valparaíso: Rarius in glareosis ad confluentes Rio Colorado et Rio Chille, Dec, *E. Poeppig 2* [12, Diar. 562] (lectotype designated here: HAL! [135744]; isolectotypes: B [destroyed, F! neg. 3057], P! [P00477033]).

#### Taxonomic notes.

Grau and Gronbach (1984: 147) erroneously accepted as the first valid publication of this species that of Bentham (1846). Consequently, they chose as a type a specimen from G-DC, linking it to the original publication. Regarding this matter, it is important to say that we have not seen this specimen, as it is not available at Global Plants JSTOR (https://plants.jstor.org/) or at the herbarium catalogue of the institution (http://www.ville-ge.ch/musinfo/bd/cjb/chg/). *Schizanthus alpestris* was described and validly published almost 20 years earlier by Poeppig (1829). Hence, the type cited by Grau and Gronbach (1984) is not that of Poeppig’s name.

Poeppig (1829) does not mention a type specimen or a specific area of collection, but the title of his work refers to collections made at Río Colorado in the Andes of Chile and being gathered by him on 24^th^ of December of 1827. We have found two specimens that can be related to this trip, but none of them are part of the Poeppig herbaria at W or at B and LE, where his samples were distributed by G. Kunze (Stafleu and Cowan 1983). However, the sheets at HAL [135744] and P [P00477033] have a printed label, where the original publication is mentioned (Not. XXIII); these were distributed after Poeppig described the species. Hence, we think these samples are part of the material used in the description, and thus original material. As no holotype is mentioned in the description and we found two specimens referring to the same collection, a lectotypification is made here. Our selection of the lectotype was based on the best-preserved specimen that shows the morphological characters of the species and the full locality data. According to the printed label on the specimens, the material was collected at the confluence of the rivers Colorado and Chille. The latter corresponds to an old name for the Aconcagua River (Risopatrón 1924), area which agrees with the currently known distribution of the species.

A further two specimens collected by Poeppig were not considered as type material because of the following reasons: the locality cited on the specimen at P [P00477034] is insufficient to be linked to the protologue (“*Chile boreal. Andes.*”). On the other hand, the specimen at F [875198] has two labels: one of them contains the locality and date of collection (“*Andes de Sa. Rosa. Chile. 1828*”), data which do not agree the protologue. A second label refers to two different numbers given to the species on Poeppig’s diary (“*N° 12. Schizanthus alpestris Pg. Diar. 562. A.*”). Those numbers are not to be associated to numbers of collection, which is different.

#### Key characters.

Delicate small flowers (14–18 mm long, 14–16 mm wide), external portion of the corolla lavender or lilac, upper middle corolla lobe narrowly oblong, with a bilobed apex, sometimes these lobes a little divided.

#### Distribution.

Endemic to Chile, between the Regions of Atacama (Province of Huasco, 28°50' lat. S) and Valparaíso (Province of Los Andes, 32°50' lat. S). 180–2800 m a.s.l.

#### Habitat.

*Schizanthus alpestris* is abundant on stony hillsides, along railroads, roadsides, and watercourses; among rocks over loose soil. It has also been found in rocky areas, where it seems to be scarce. In these rocky areas, it grows with *Puya* sp. (Bromeliaceae), *Flourensia thurifera* DC. (Asteraceae), *Echinopsis chiloensis* (Colla) H.Friedrich and G.D.Rowley (Cactaceae) and *Proustia cuneifolia* D.Don (Asteraceae).

#### Conservation.

**Chile. Coquimbo**: Las Chinchillas National Reserve.

#### Selected specimens examined.

**Chile. Atacama: [Huasco Province**] Incahuasi, 25 kms al sur, 10 Oct 1958, *M. Ricardi & C. Marticorena 4902/1287* (CONC); Norte entrada de Los Cristales, cuesta ruta 5 entre Vallenar y llanos La Higuera, en ladera rocosa del cerro, 29°9'18.5"S, 70°59'57.9"W, 1010 m a.s.l., 8 Oct 2008, *M. Muñoz 5049* (SGO); **Coquimbo: [Elqui Province**] Camino Paihuano – Rivadavia, 840 m a.s.l., 28 Sep 1948, *F. Behn s.n.* (CONC); Camino de Marquesa a Condoriaco, 3 kms antes de Talcuna, 500 m a.s.l., 18 Oct 1971, *C. Marticorena*, *R. Rodríguez & E. Weldt 1514* (F); Cuesta de Andacollo, en mitad de la cuesta, 16 Sep 1957, *C. Muñoz 4281* (SGO); [**Limarí Province**] Camino al Embalse de La Laguna, a 16 km del Embalse, 2500 m a.s.l., 5 Feb 1963, *M. Ricardi*, *C. Marticorena & O. Matthei 730* (CONC); Pulpica, 30°53'S, 70°46'W, 9 Mar 2008, *Fundación Philippi 366* (SGO); Combarbalá, 1000 m a.s.l., Sep 1936, *C. Grandjot s.n.* (SI); [**Choapa Province**] Cerro Gonzalo, cara N y W, 32°1'S, 71°6'W, 1740 m a.s.l., 5 Dec 2006, *M. Rosas & M. Acosta 4294* (CONC); Cuesta El Espino, 31°21'39.2"S, 71°5'51.7"W, 1260 m a.s.l., 9 Oct 2004, *M. Muñoz 4473* (SGO); **Valparaíso: [Petorca Province**] Petorca – Quebrada El Durazno, 32°12'7.58"S, 71°0'44.71"W, 1490 m a.s.l., 7 Nov 2017, *J. Macaya*, *S. Teillier*, *P. Novoa & O. Fernández 370* (CONC); In Chincolco, 1500 m a.s.l., 28 Dec 1972, *O. Zoellner 6741* (L); Las Tasas, Cajón del Pedernal, 23 Aug 1894, *P. Germain s.n.* (SGO); [**Quillota Province**] Cerro Caquis, ca. 15 km east of Melon, 1800 m a.s.l., 14 Dec 1938, *J.L. Morrison 16879* (SI); Cordillera El Melón, subida al cerro Mosco Verde, cerca cumbre, 32°39'6"S, 71°2'50"W, 2049 m a.s.l., 18 Jan 2011, *A. Moreira 1423* (SGO); [**San Felipe Province**] En el cerro Orolongo bei San Felipe, 2000 m a.s.l., 19 Nov 1972, *O. Zoellner 6210* (L); [**Los Andes Province**] Aconcagua, Los Maitenes (Río Colorado), 2000 m a.s.l., 18 Nov 1970, *O. Zoellner 4434* (CONC); In Maitenes bei Río Colorado, 2200 m a.s.l., 18 Nov 1970, *O. Zoellner 4462* (L).

### 6. Schizanthus laetus

Taxon classificationPlantaeSolanalesSolanaceae

Phil., Fl. Atacam.: 45. 1860

F238C5E2-E1B6-5053-AD3B-1B93337A7902

Fig. 3E–H

Schizanthus fallax I.M.Johnst., Contr. Gray Herb. 85: 160. 1929.

#### Type.

Chile. Antofagasta: Cachinal, *R.A. Philippi s.n*. (lectotype designated by Grau and Gronbach 1984, pg. 146 [as type]: SGO! [SGO000004526 acc. #055389]).

#### Taxonomic notes.

Johnston (1929) characterised *S. fallax* as representing the northern-most distribution of the genus and having the upper lateral lobes of the corolla larger and not deeply lobed as in *S. laetus*. Grau and Gronbach (1984: 146) considered the characters of *S. fallax* as part of the variability and their broad concept of *S. laetus*, citing the name by Johnston (1929) as a synonym. We have revised the type material of *S. fallax* through Global Plants JSTOR (https://plants.jstor.org/) (Chile. Antofagasta: Tocopilla, steep hillside ca. 6 km. north of port and about opposite Caleta Duendes, 18 Oct 1925, *I.M. Johnston 3626* (holotype: GH! [00077406]; isotypes: F! [F0073042F acc. #625799], K! [K000585355, photo at IND! [IND-0107172]], S! [acc. #S04-3140], US! [00028096 acc. #1473978]). Some of these specimens suggest that *S. fallax* may represent a distinct species, but we do not have access to additional material and prefer to maintain the synonymy pending further study.

#### Key characters.

Flowers dark violet or paler, upper middle lobe without a distinctive yellow area, but white and dotted with dark spots at the base. Lower lip of the corolla of similar length as the upper lip.

#### Distribution.

Endemic to Chile, in the coast of the Regions of Tarapacá (Province of Iquique, 20°40' lat. S) and Antofagasta (Province of Antofagasta, 26°0' lat. S). 20–900 m a.s.l.

#### Habitat.

*Schizanthus laetus* grows in the fog (camanchaca) zone, on steep hillsides, in watercourse and alluvial fans; between rocks and in sandy and gravel soil (coarse sand). Within these places, it prefers wet areas with organic material. Forms part of Lomas vegetation where it can be associated with *Echinopsis deserticola* (Werderm.) H.Friedrich and G.D.Rowley (Cactaceae) and *Euphorbia lactiflua* Phil. (Euphorbiaceae).

#### Conservation.

**Chile. Antofagasta**: Paposo Norte Natural Monument, Pan de Azúcar National Park (Rundel et al. 1996).

#### Selected specimens examined.

**Chile. Tarapacá: [Iquique Province**] Camino Iquique a Patillos, cumbres de los cerros frente al km 22, 17 Oct 1965, *M. Ricardi*, *C. Marticorena & O. Matthei 1343* (CONC); Alto Punta Lobos, 21°2'S, 70°9'W, 430 m a.s.l., 1 Nov 1997, *R. Pinto s.n.* (SGO); **Antofagasta: [Tocopilla Province**] Tocopilla, steep hillside ca. 6 km north of port and approximately opposite Caleta Duendes, 18 Oct 1925, *I.M. Johnston 3626* (F, GH, IND, K, US); 1 km al N de la Planta Mantos de La Luna, a lo largo de la ruta costanera, ca. 5 km al N de caleta Buena, 22°22'27"S, 70°13'73"W, 150–300 m a.s.l., 18 Oct 2002, *J.V. Schneider & M.L. Huertas 2851* (CONC); Cobija, Quebrada Aguada Cañas, 500–800 m a.s.l., 4 Dec 1949, *W. Biese 3080* (SGO); [**Antofagasta Province**] Quebrada de Miguel Díaz, en Punta Miguel Díaz, suelo arenoso, ca. 350 m a.s.l., 12 Oct 1941, *E. Pisano & R. Bravo 453* (CONC); Quebrada Miguel Díaz, en Punta Miguel Díaz, 350 m a.s.l., 12 Oct 1941, *E. Pisano & R. Bravo 453* (SGO); Quebrada Guanillo (10 km al N del Cachinal de la costa), 50–500 m a.s.l., 14 Dec 1949, *W. Biese 3303* (SGO); Paposo, entrada a la Qda. Los Peralitos, 25°1'57"S, 70°26'30.3"W, 490 m a.s.l., 30 Sep 2005, *M. Muñoz 4606* (SGO).

### 7. Schizanthus pinnatus

Taxon classificationPlantaeSolanalesSolanaceae

Ruiz & Pav., Fl. Peruv. [Ruiz & Pavon] 1: 13. 1798

A9C34FF1-BB2A-59C4-8C54-2BDA2CBD67A7

Fig. 3I–L

Schizanthus pinnatus β ? gracilis Benth., Prodr. [A.P. de Candolle] 10: 202. 1846.
Schizanthus gracilis (Benth.) Clos, Fl. Chil. [Gay] 5(2): 153. 1849.
Schizanthus gayanus Phil., Linnaea 30(2): 198. 1859.
Schizanthus latifolius Phil., Linnaea 33(2): 214. 1864.
Eutoca pedunculosa Phil. Anal. Univ. Chile 65: 61. 1884.
Schizanthus humilis Phil., Anal. Univ. Chile 91: 118. 1895.
Schizanthus floribundus Phil., Anal. Univ. Chile 91: 119. 1895.

#### Type.

Chile. Unknown: Chili, *J. Dombey s.n.* (lectotype designated by Grau and Gronbach 1984, pg. 139 [as type]: BM! [BM000992219]).

#### Taxonomic notes.

This species was described on Ruiz and Pavón (1798) using the samples collected in Chile, during the botanical expedition to the Viceroyalty of Peru. The expedition to the Chilean territory was undertaken from 27^th^ January of 1782 until October of 1783 with the participation of Hipólito Ruiz, José Pavón and Joseph Dombey (Marticorena 1995).

Regarding the type material, the protologue clearly states that the authors of the species had access to material from different collections: “*Esquadron*” [Escuadrón] located in the Municipality of Coronel and “*Araucanía*”, referring to an area they visited. Therefore, the samples matching the protologue should be recognised as syntypes, requiring lectotypification.

We have found 11 herbarium sheets in five herbaria that correspond to this expedition (BM [BM000992219, BM000994723], F [V0126154F], MA [MA 815287, MA 815288, MA 815289], L [L.2881253], P [P00477581, P00477582, P00477583, P00675638]). Most of the sheets mentioned only the country of origin, except for the specimens at MA [MA 815287] and P [P00477583], which mentioned a more detailed locality: “*In Coronel juxta Concepcion*” and “*Concepcion*”, respectively. The specimen at MA [MA 815287] gives a date (February) that agrees to the time when the collectors visited the localities mentioned in the protologue. It is also accompanied by the handwritten description and some drawings of the species. This specimen was identified as lectotype by F. Bellot in 1974 but he never appropriately published his findings. Later, the specimen at BM was cited as the lectotype of the species by Grau and Gronbach (1984: 139), as it matches the protologue and what is known about the possible collectors (“*Chili*, *Dombey*”). We are not designating any of the additional 10 specimens we have seen as isolectotypes, because the lack of data on their labels does not allow us to verify if any of them are duplicates of the lectotype.

We do not know why Grau and Gronbach (1984) chose the specimen at BM over the sheet at MA, where most of the types by Ruiz and Pavón are deposited (Stafleu and Cowan 1983). We have examined Turland et al. (2018, Art. 9.19.), without finding valid arguments for replacing the lectotypification made by Grau and Gronbach (1984).

#### Key characters.

This species has small flowers (16–20 mm long, 14–15 mm wide), with the lower lip a little longer than the upper lip. Corolla colour from purple to white, upper middle lobe oblong, white or pale yellow at the base, this area dotted with dark spots and surrounded by a regular or irregular purple stripe (sometimes absent). The upper lateral lobes also have dark spots, which are sometimes faint.

#### Distribution.

Endemic to Chile, between the Regions of Valparaíso (Province of San Antonio, 33°35' lat. S) and Los Lagos (Province of Osorno, 40°30' lat. S). 30–2000 m a.s.l.

#### Habitat.

*Schizanthus pinnatus* can be found on dry hillsides and alluvial terraces of native forest and matorrals; in some areas it grows under plants of *Acacia caven* (Molina) Molina (Fabaceae). Abundantly in harvested plantation of *Pinus radiata* D.Don (Pinaceae).

#### Conservation.

**Chile. Metropolitana**: Altos de Cantillana Natural Sanctuary; **O’Higgins**: Alto Huemul Natural Sanctuary; **Maule**: Radal Siete Tazas National Park, Altos de Lircay National Reserve, Bellotos del Melado National Reserve, Cajón del Río Achibueno Natural Sanctuary (Bravo Monasterio et al. 2014), Humedal de Reloca Natural Sanctuary; **Ñuble**: Los Huemules de Niblinto National Reserve (Rodríguez et al. 2008); **Araucanía**: Nahuelbuta National Park.

#### Selected specimens examined.

**Chile. Valparaíso: [San Antonio Province**] Rocas de Santo Domingo, Oct 1956, *G. Galingo s.n.* (CONC); **Metropolitana: [Maipo Province**] Cerro Challay, 2 Nov 1993, *R. Peña 840* (SGO); **O’Higgins: [Cachapoal Province**] Rancagua, old road to Termas de Cauquenes S of Río Cachapoal, ca. 12 km E of Hwy. 5 (Panamericana), 34°15'S, 70°45'W, 6 Oct 1993, *L.R. Landrum & S.S. Landrum 7900* (ASU); Cocalán (lad. exp. N), coord. UTM 300777E – 6213646N, 197 m a.s.l., 22 Nov 2005, *N. García, F. Romero & P. Contreras 2948* (CONC); Ex Laguna de Tagua-Tagua, Valle o Rinconada de Huinca, desde las casas hasta 200 m alt, 10 Nov 1967, *M. Muñoz 156* (SGO); [**Cardenal Caro Province**] El Espinillo, Poza El Encanto (ladera norte), coord. UTM 236051E – 6177258N, 33 m a.s.l., 29 Oct 2005, *N. García, F. Romero & P. Contreras 2368* (CONC); Tanumé, Quebrada Honda (ladera exposición norte), coord. UTM 232675E – 6213295N, 262 m a.s.l., 25 Oct 2005, *N. García, F. Romero & P. Contreras 2178* (CONC); [**Colchagua Province**] San Fernando, Fundo Los Alpes, Río Antivero, 26 Nov 1949, *T. Gutiérrez 54* (SGO); Yaquil (plano inclinado exp. S), coord. UTM 299844E – 6167762N, 277 m a.s.l., 18 Nov 2005, *N. García, F. Romero & P. Contreras 2740* (CONC); **Maule: [Curicó Province**] Reserva Nacional Radal Siete Tazas, 35°26'870"S, 71°2'298"W, 803 m a.s.l., 11 Dec 2004, *A. Marticorena*, *A. Jiménez & A. Pauchard 37* (CONC); [**Talca Province**] Alto de Vilches, Laguna El Alto, a orilla de la laguna, 35°37'S, 71°1'W, 2050 m a.s.l., 31 Jan 2000, *V. Finot & P. López 1886* (CONC); Constitución, 19 Oct 1942, *R. Silva 224* (SGO); [**Linares Province**] Tranque Bullileo, 600 m a.s.l., 11 Jan 1952, *P. Aravena 67* (SGO, US); [**Cauquenes Province**] Santuario Reloca, 35°39'S, 72°35'W, 40 m a.s.l., 1 Oct 1999, *M.T.K. Arroyo*, *J. Armesto*, *A.M. Humaña*, *F. Pérez*, *D. Rougier & R. Guevara 992712* (CONC); **Ñuble: [Diguillín Province**] Palomares, 36°34'4.23"S, 72°36'34.69"W, 57 m a.s.l., Nov 2008, *A. Marticorena s.n.* (CONC); Río Chillán, 36°38'S, 72°4'W, 120 m a.s.l., 2 Dec 2008, *N. García 4332* (CONC); [**Itata Province**] Huechupín, Mar 1864, *M. de Solís s.n.* (SGO); **Biobío: [Concepción Province**], San Pedro, 18 Dec 1960, *C.S. Takayama s.n.* (CONC); [**Biobío Province**] Antuco, 21 Dec 1964, *G. Montero s.n.* (CONC); Los Ángeles, Antuco, 21 Dec 1964, *G. Montero 7052* (IND); Nacimiento, Fundo El Tambillo, 12 Nov 1950, *A. Pfister s.n.* (CONC); [**Arauco Province**] Laraquete, 15 Dec 1950, *A. Pfister & M. Ricardi s.n.* (CONC); Arauco, Dec 1927, *Bro. Claude-Joseph 5613* (US); **Araucanía: [Malleco Province**] Mininco, 10 Nov 1953, *H. Gunckel 2285* (CONC); Fuera del Parque Nacional Nahuelbuta, 1 Dec 1987, *I. Meza & E. Barrera 1651* (SGO); Fundo Solano, Los Alpes, Cordillera de Nahuelbuta, 1200 m a.s.l., 18 Jan 1958, *W.J. Eyerdam 10347* (US); [**Cautín Province**] Pucón – Lago Villarrica, Feb 1935, *A. Pfister s.n.* (CONC); **Los Ríos: [Valdivia Province**] Quinchilca, 70 m a.s.l., 20 Jan 1942, *A. Hollermayer 557* (SGO); Puñire [Panguipulli], ca. 200 m a.s.l., Feb 1925, *A. Hollermayer 694* (CONC, E, F, L, SI, US); [**Del Ranco Province**] Daglipulli, Feb 1835, *C. Gay 176* (P); **Los Lagos: [Osorno Province**] In sepiis rarissimus Osorno, Mar 1835, *C. Gay 177* (P); Ad flumen Pilmaiquen, *D. Cueto s.n.* (SGO).

### 8. Schizanthus hookeri

Taxon classificationPlantaeSolanalesSolanaceae

Gillies ex Graham, Edinb. N. Phil. Journ. 9: 176. 1830

B66D2CF2-8229-5B83-A11D-C6A84B9D9C45

Fig. 4A–D

Schizanthus calycosus Phil., Anal. Univ. Chile 43: 529. 1873.
Schizanthus hookeri var. calycosus (Phil.) Reiche, Anal. Univ. Chile 125: 480. 1909.

#### Type.

Chile. Unknown: In various places on the Chilean side of the Cordillera of the Andes, 8000 ft alt., *J. Gillies s.n.* (neotype designated by Grau and Gronbach 1984, pg. 128 [as type]: K! [K000648571, photo at E! [E00089590], IND! [IND-0107179]]).

**Figure 4.**
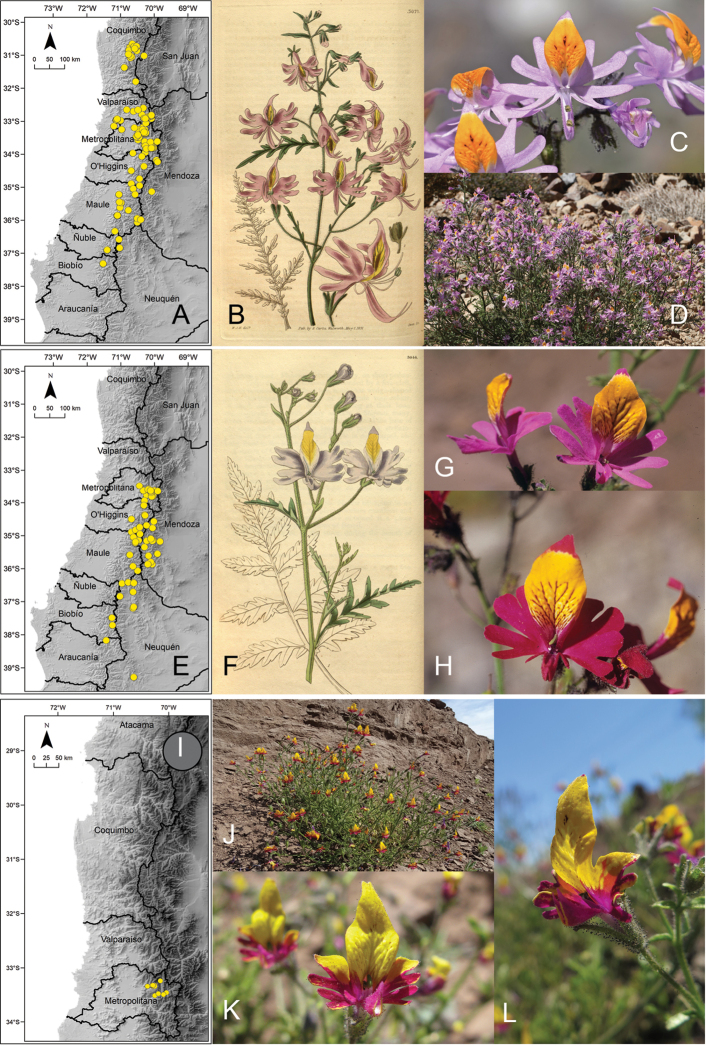
**A** distribution of *Schizanthus hookeri***B** illustration of *S. hookeri* published with the description of the species (Graham 1830) **C** examples of *Schizanthus hookeri* in Juncal Andean Park **D** La Campana National Park **E** distribution of *Schizanthus grahamii***F** illustration of *S. grahamii* published with the description of the species (Hooker 1831a) **G** examples of *S. grahamii* in Paso Vergara **H** Termas del Flaco **I** distribution of *Schizanthus coccineus***J–L** examples of *S. coccineus* in La Parva (Metropolitan Region). Photos by M. Aldunate (**C**), A. Moreira-Muñoz (**D, K**), S. Moreira (**G, H**), C. Jirón (**J**), V. Morales (**L**).

#### Taxonomic notes.

According to the protologue, the species was described from samples grown at the private garden of Mr. Boog, in Portobello, close to Edinburgh (Scotland). These plants were raised from the seeds collected by Gillies “*in various places on the Chilian side of the Cordillera of the Andes*, *at an elevation of 8000 or 9000 feet above the level of the sea*” (Graham 1830: 177). At the very end of the description, Graham says that he received a letter by Gillies with a list of characteristic features of this new species and a specimen, but he does not mention the origin of the sample (from cultivation or from the wild).

During our search, we have not found cultivated samples of the species seen by Graham but one specimen at E [E00089582] collected by Gillies in “*Andes of Chile et Mendoza*”. As in other cases, we cannot be sure if this sample was seen or used by Graham before or while he wrote the description. Therefore, we do not consider this specimen as original material.

Grau and Gronbach (1984), cited as type of *S. hookeri* a sample at K [K000648571], which exhibits the protologue information on the label (where the seeds were collected). In this case, it is possible that the data on the label could be copied from the protologue. Here we recognise this specimen as a neotype because we could not find original material. The same specimen at K has been cited as a holotype by Cosa (2013: 316), but given the previous reason, this should be considered incorrect.

#### Key characters.

Delicate and slender flowers, corolla lilac to rose, with stamens protruding from the corolla tube. The lower middle lobe attenuated into two caudate apex.

#### Distribution.

Southern Andean, endemic from Argentina and Chile. In the mountains of the Coastal range and the Andes. In Chile it grows from Coquimbo (Province of Limarí, 30°35' lat. S) to Biobío (Province of Biobío, 37°20' lat. S) and in Argentina it occurs in the Provinces of Mendoza and Neuquén. 900–3200 m a.s.l.

Cosa (2013: 317) rejects the presence of this species in the Argentinian Province of Mendoza, after checking specimens collected within the limits of this administrative area. However, we have found one sample of this taxon in the Department of Malargüe (Mendoza) (see the selected specimens examined).

#### Habitat.

*Schizanthus hookeri* is abundant in well-drained screes, along stream sides, slopes next to roads and moraines. Within these places, it grows in fine sandy soil, among rocks. It seems to be less frequent in open areas, including sunny banks or north-facing slopes. Regarding the vegetation, it can be seen in forest of *Nothofagus* Blume, arborescent scrub of *Chuquiraga oppositifolia* D.Don (Asteraceae), *Baccharis neaei* DC. (Asteraceae) and *Festuca acanthophylla* É.Desv. (Poaceae).

#### Conservation.

**Argentina. Neuquén**: Epu-Lauquén Protected Natural Area.

**Chile. Valparaíso**: La Campana National Park, Río Blanco National Reserve, Serranía El Ciprés Natural Sanctuary, Juncal Andean Park; **Metropolitana**: El Morado Natural Monument, Cerro El Roble Natural Sanctuary, Yerba Loca Natural Sanctuary; **Maule**: Radal Siete Tazas National Park, Altos de Lircay National Reserve, Bellotos del Melado National Reserve; **Ñuble**: Los Huemules de Niblinto National Reserve (Rodríguez et al. 2008); **Biobío**: Laguna del Laja National Park.

#### Selected specimens examined.

**Argentina. Mendoza: [Malargüe Department**] Trayecto desde Malargüe a Las Loicas, 1400–1750 m a.s.l., 29 Jan 1994, *C. Villagrán*, *F. Hinojosa & R. Villa 8108* (CONC); **Neuquén: [Huilliches Department**] Reserva Laguna de Epu-Lauquen, 36°49'3"S, 71°4'51"W, 1470 m a.s.l., 2 Dec 2012, *F.O. Zuloaga*, *L. Aagesen*, *M.V. Nicola & D.L. Salariato 15173* (SI);

**Chile. Coquimbo: [Limarí Province**] El Maitén, 90 km al oriente de Ovalle, 1340 m a.s.l., 13 Nov 1943, *R. Wagenknecht 149* (CONC, SGO, US); Cord. Ovalle, Cerro Loica, 2200 m a.s.l., 18 Dec 1965, *C. Jiles 4734* (CONC); Camino a Central Los Molles, km 13, 30°43'42.4"S, 70°31'28.6"W, 1700 m a.s.l., 21 Jan 2005, *M. Muñoz 4535* (SGO); [**Choapa Province**] Cerro Curimahuida, 10 km east of Matancilla and 15 km northeast of Sánchez mine, 2600–2800 m a.s.l., 23 Nov 1938, *C.R. Worth & J.L. Morrison 16671* (SI); Dpto. Illapel, La Polcura, 31°30'S, 70°40'W, 2400 m a.s.l., 16 Feb 1962, *C. Jiles 4255* (CONC); Las Mollacas, Cord. d. Illapel, Jan 1888, *Unknown s.n.* (SGO); **Valparaíso: [Quillota Province**] Between abandoned copper mine and bronze memorial plaque to Darwin, near summit of La Campana (Bell Mountain), 10 miles east of El Granizo, ca. 1750 m a.s.l., 15 Dec 1957, *W.J. Eyerdam 10087* (F, SGO, US); Above Ramayama copper mine, Cerro Las Vizcachas, 1860 m a.s.l., 7 Dec 1951, *P.C. Hutchinson 95* (F, US); Cerro La Campana, cerca de placa de Darwin, 1600 m a.s.l., 25 Nov 1962, *P. Weisser 390* (CONC); [**San Felipe Province**] Santuario Serranía El Ciprés, 32°39'12"S, 70°48'57"W, 1779 m a.s.l., 21 Dec 2013, *A. Madrid & J. Larraín 186* (CONC); [**Los Andes Province**] Portillo, Caracoles, 32°50'S, 70°7'W, 2580 m a.s.l., 14 Feb 1995, *K. Gengler 35* (CONC); Parque Andino Juncal, Sendero Las Canchitas, 32°55'17.5"S, 70°4'58.1"W, 2726 m a.s.l., 5 Mar 2011, *M.F. Gardner*, *C. Morter & G. Ovstebo 273* (E); Minera Andina, Saladillo, bajando desde túnel principal, 33°3'41.5"S, 70°14'54.6"W, 2900 m a.s.l., 16 Jan 2002, *M. Muñoz 4147* (SGO); **Metropolitana: [Chacabuco Province**] Altos de Chicauma, 33°12'S, 70°56'W, 2050 m a.s.l., 10 Jan 2003, *N. García 3799* (CONC); Camino a cerro El Roble, portezuelo hacia mina de cuarzo, 11 Jan 2004, *M. Muñoz 4413* (SGO); Estero Colina, en el lecho mayor del estero, 33°11'56.5"S, 70°35'30"W, 14 Nov 2009, *V. Morales & F. Cornejo 9* (SGO); [**Santiago Province**] Santuario de la Naturaleza Yerba Loca, ladera al NO del estero de La Yerba Loca, sector Qda. Agua Blanca, 33°17'S, 70°19'W, 2430 m a.s.l., 27 Feb 2000, *M.T.K. Arroyo*, *M. Mihoc & C. Valdivia 202026* (CONC); Valle Nevado, 3 Km del cruce con La Parva, 33°21'34.5"S, 70°17'34.2"W, 2380 m a.s.l., 25 Jan 2003, *M. Muñoz 4352* (SGO); Peñalolén, 1600–2300 m a.s.l., 30 Dec 1928, *G. Looser 914* (SI); [**Cordillera Province**] San José de Maipo, Cajón de Morales: entre Baños Morales y las Panimávidas, 33°48'S, 70°4'W, 1850 m a.s.l., 22 Dec 2001, *S. Teillier & C. Márquez 5255* (CONC); Bei Lo Valdés, 2500 m a.s.l., 2 Jan 1968, *O. Zoellner 2561* (L); Cajón del Yeso, Termas El Plomo, 3000 m a.s.l., 20 Jan 1995, *M. Muñoz*, *A. Moreira*, *I. Meza & J. Arriagada 3589* (SGO); **O’Higgins: [Cachapoal Province**] Camino de Caletones a Colón, km 2, 1700 m a.s.l., 17 Nov 1970, *C. Marticorena & E. Weldt 661* (CONC, F); Cajón del Río Claro, Rengo, 34°30'S, 70°41'W, 6 Nov 2003, *Fundación Philippi 97* (SGO); [**Colchagua Province**] Quebrada camino V. del Flaco, Huertecillas, 1100 m a.s.l., 8 Jan 1951, *M. Ricardi s.n.* (CONC); Alto Huemul, coord. UTM 345976E – 6137905N, 1645 m a.s.l., 3 Jan 2006, *L. Faúndez & B. Larraín 1289* (CONC); **Maule: [Curicó Province**] Hacienda Monte Grande, ca. 1700 m a.s.l., Dec 1924, *E. Werdermann 509* (CONC, E, F, SI, U, US); Molina, Área de Protección, Radal Siete Tazas, camino hacia cerro El Alto, 29 Dec 1989, *M. Muñoz 2514* (SGO): [**Talca Province**] Laguna del Maule, 36°1'S, 70°33'W, 2250 m a.s.l., 24 Jan 1990, *M.F. Gardner & S. Knees 4473* (E, SGO); Alto de Vilches, camino a Laguna El Alto, 35°36'S, 71°1'W, 1800 m a.s.l., 29 Jan 2000, *V. Finot & P. López 1732* (CONC); [**Linares Province**] Reserva Nacional Bellotos del Melado, 35°51'S, 71°5'W, 1330 m a.s.l., 19 Dec 1999, *M.T.K. Arroyo*, *P. MacPherson*, *M. Mihoc*, *A. Humaña & C. Valdivia 996027* (CONC, SGO); Cajón de Ibáñez, 19 Jan 1938, *Castellanos s.n.* (BA); **Ñuble: [Diguillín Province**] Termas de Chillán, 1800 m a.s.l., 5 Feb 1936, *A.L. Cabrera 3634* (F); Termas de Chillán, Sendero hacia fumarolas, sobre nivel del bosque, 6 Feb 1993, *M. Muñoz 3239* (SGO); Cord. de Chillán, Los Moscos, 1700 m a.s.l., Jan 1937, *C. Grandjot & G. Grandjot 2028* (CONC); **Biobío: [Biobío Province**] Orillas de la Laguna del Laja, 24 Jan 1969, *M. Ricardi & C. Marticorena 5816/1977* (CONC, F).

### 9. Schizanthus grahamii

Taxon classificationPlantaeSolanalesSolanaceae

Gillies ex Hook., Bot. Mag. 58: tab. 3044. 1831

7635BBA4-E3EB-561B-B6DF-FEC96DFBFA3B

Fig. 4E–H

Schizanthus retusus Hook., Bot. Mag. 58: tab. 3045. 1831.
Schizanthus gilliesii Phil., Linnaea 29(1): 28. 1858.
Schizanthus araucanus Phil., Anal. Univ. Chile 91: 121. 1895.
Schizanthus diazii Phil., Anal. Univ. Chile 91: 122. 1895, as “*diazi*”.
Schizanthus grahamii var. araucanus (Phil.) Reiche, Anal. Univ. Chile 125: 478. 1909.

#### Type.

Argentina. Mendoza: On the Mendoza side of the cordillera of the Andes at an elevation of about 9000 feet, *J. Gillies s.n.* (neotype designated by Grau and Gronbach 1984, pg. 124 [as type]: K! [K000585353]).

#### Taxonomic notes.

The original description mentions that the species was grown in the private garden of Mr. Boog in Portobello, raised from seeds collected by Gillies in Chile. Together with the description, an illustration by Dr. Greville was published.

The species was described and validly published by W.J. Hooker in 1831, while he was working as a professor of botany in Glasgow. Therefore, the material seen by him should be found at GL (now on permanent loan to E), although, some of his types were moved to K when he was appointed director of the Royal Botanic Gardens, Kew (Stafleu and Cowan 1979). We have searched for cultivated specimens of the species on these herbaria and we have not found specimens that could be linked to the protologue. However, we have found a cultivated specimen at GH [00077407], labelled at the top right corner as “*S. grahamii Gillies*”. The smaller branch on the sheet, located at the right bottom corner says “*Mr. Boog’s Garden Portobello 30^th^ July 1830*”. In this case, the place and date of flowering corresponds with the data given in the original description. The other three branches on the sheet were labelled as “*Native specimens from Dr. Gillies 26 Augt 1830*”. We think this refers to the date of collection at Portobello, because Gillies was in South America until 1828. In our opinion, the specimen at GH cannot be considered as original material because we cannot be sure if it was seen by the author of the species.

Here we accept the inadvertent neotypification made by Grau and Gronbach (1984: 128), as they selected a specimen of the species that was collected by Gillies in the Andes of Mendoza [K000585353]. This selection does not look obvious as the protologue mentions Chile as the place where the seeds were collected. However, at the time when Gillies collected the material, Mendoza was associated with the administrative area called Corregimiento de Cuyo, controlled by the Capitanía General de Chile. In a previous publication, Cosa (2013: 316) cited the same sample at K as holotype of *S. grahamii*. We think this is not correct, as the holotype of the species corresponds to a cultivated sample.

#### Key characters.

The upper middle corolla lobe is almost completely yellow, except for the apex and it looks bigger than the other segments of the corolla. Often confused with *S. hookeri*, because of its geographical distribution and the corolla form. However, the flower has short stamens, that barely protrude from the corolla tube and the lower middle lobe is attenuated into two short pointed apex.

#### Distribution.

Southern Andean, endemic from Argentina and Chile. In Chile it grows from the Metropolitan Region (Province of Santiago, 33°25' lat. S) to Biobío, while on the Argentinian side it inhabits the Provinces of Mendoza and Neuquén (Department of Catan Lil, 39°20' lat. S). 1200–2900 m a.s.l.

#### Habitat.

*Schizanthus grahamii* is abundant in areas close to watercourses, such as lakes, rivers or quebradas. It grows among rocks or in loose stony places, also located at the bases of hillsides, where it is common to find scree slopes and alluvial plains. It seems to grow very well in shady places (south-facing slopes), dominated by *Acaena splendens* Hook. & Arn. (Rosaceae), *Calceolaria hypericina* Poepp. ex Benth. (Calceolariaceae), *Calceolaria dentata* Ruiz & Pav. (Calceolariaceae), *Glandularia berteroi* (Schauer) Muñoz-Schick (Verbenaceae) and in places by thickets of *Diostea juncea* (Gillies & Hook. ex Hook.) Miers (Verbenaceae) and among low shrubby vegetation.

#### Conservation.

**Argentina. Neuquén**: Epu-Lauquén Protected Natural Area.

**Chile. Metropolitana**: Río Clarillo National Reserve, El Morado Natural Monument; **Biobío**: Laguna del Laja National Park (Rondanelli et al. 2000).

#### Selected specimens examined.

**Argentina. Mendoza: [Malargüe Department**] De Ruta Nacional 40 a Valle de Las Leñas, 35°10'36"S, 69°49'60"W, 22 Nov 2010, *F.O. Zuloaga*, *D.L. Salariato*, *C.A. Zanotti & L. Zavala 12337* (SI); RP 226, de Las Loicas a termas del Azufre, camino hacia las termas, 35°20'56"S, 70°17'44"W, 2002 m a.s.l., 19 Jan 2018, *D.L. Salariato*, *L. Aagesen*, *J.M. Acosta & A. Martínez 100* (SI); **Neuquén: [Huiliches Department**] Lagunas de Epu-lauquén, 36°49'8"S, 71°3'10"W, 1520 m a.s.l., 26 Nov 2010, *F.O. Zuloaga*, *D.L. Salariato*, *C.A. Zanotti & L. Zavala 12517* (SI) [**Minas Department**] Laguna Varvarco Campos, 36°25'27"S, 70°37'9"W, 1959 m a.s.l., 15 Feb 2007, *J. Chiapella*, *G.E. Barbosa*, *F. Chiarini & M. Matesevach 1865* (SI);

**Chile. Metropolitana: [Cordillera Province**] Along the Embalse El Yeso, along the access road 4 km upstream from the dam, 33°35–40'S, 70°10–15'W, 2510 m a.s.l., 14 Jan 1993, *C.M. Taylor & R.E. Gereau 10927* (ASU, CONC, SGO); Río Volcán – Cajón del Morado, 33°46'41"S, 70°2'32"W, 2580 m a.s.l., 29 Jan 2009, *S. Teillier*, *F. Romero*, *I. Goic & X. Romero 5628A* (CONC); Laguna Negra ribera Este, 33°38'51"S, 70°6'46"W, 2916 m a.s.l., 5 Dec 2008, *A. Moreira 1107* (SGO); **O’Higgins: [Cachapoal Province**] El Teniente, rock-slides, near Río Coya, 2500–2700 m a.s.l., 26 Jan 1925, *W. Pennell 12282* (F, SGO); Cajón del Río Claro, Rengo, 34°30'S, 70°41'W, 6 Nov 2003, *Fundación Philippi 84* (SGO); [**Colchagua Province**] Las Huertecillas, entre la Pava y Qda. San Andrés, coord. UTM 368224E – 6154259N, 1724 m a.s.l., 31 Jan 2006, *N. García*, *F. Romero & P. Contreras 3427* (CONC); Termas del Flaco, inicio y base cerro Verde hacia huellas de dinosaurios, 34°57'7.5"S, 70°25'47"W, 1790 m a.s.l., 11 Jan 2006, *M. Muñoz 4752* (SGO); **Maule: [Curicó Province**] Alrededores de la Laguna de Teno, 35°10'S, 70°33'W, 2560 m a.s.l., 29 Mar 1973, *C. Marticorena*, *O. Matthei & R. Rodríguez 19* (CONC); Camino a Paso Vergara, pasado control policial, 35°8'45"S, 70°28'29"W, 1913 m a.s.l., 28 Jan 2003, *M. Muñoz 4370* (SGO); [**Talca Province**] Por ruta nacional n115, ca. 17 km de la Laguna de Maule, viniendo desde San Clemente, 35°55'39"S, 70°38'16"W, 1424 m a.s.l., 9 Feb 2007, *J. Chiapella*, *G.E. Barbosa*, *F. Chiarini & M. Matesevach 1660* (SI); SW del Descabezado del Maule, 1877, *E. Williams s.n.* (SGO); [**Linares Province**] Laguna Dial, 35°25'S, 70°55'W, 1520 m a.s.l., 25 Jan 1961, *F. Schlegel 3671* (CONC); **Ñuble: [Punilla Province**] Cord. “San Carlos”, Feb 1925, *E. Barros s.n.* (CONC); **Biobío: [Biobío Province**] Los Pinos. Extremo sur de la Laguna Laja, 27 Feb 1951, *F. Behn s.n.* (CONC); Trapa-Trapa, Araucanía, 28 Jan 1887, *C. Rahmer s.n.* (SGO).

### 10. Schizanthus coccineus

Taxon classificationPlantaeSolanalesSolanaceae

(Phil.) J.M.Watson, Pl. Altoandinas Fl. Silv. Chile: 140. 1998

E063D862-7D46-5BD4-A245-69F5B47AD6A5

Fig. 4I–L

Schizanthus grahamii var. coccinea Phil., Anal. Univ. Chile 91: 121. 1895.

#### Type.

Chile. Metropolitana: Alfalfar, Dec 1887, *L. Kunze s.n.* (lectotype here designated: SGO! [SGO000004516 acc. #055328]; isolectotype: SGO! [SGO000004515 acc. #042891]).

#### Taxonomic notes.

In this case, we have found two sheets of the same collection that agree with the data given in the protologue. For this reason, we have designated the best preserved specimen as the lectotype.

#### Key characters.

Upper lip bi-coloured; yellow in the middle lobe and the upper half of the lateral lobes, while the lower half is mostly reddish, that continues to the lower lip. Upper middle lobe almost two times longer than the lateral lobes.

#### Distribution.

Endemic to Chile, in the Andean mountains of the Metropolitan Region (Provinces of Santiago and Cordillera, 33°15'–33°30' lat. S). 2000–2900 m a.s.l.

#### Habitat.

*Schizanthus coccineus* has been found growing over loose soil; mostly along roadsides and in wet or flooded areas (vegas).

#### Conservation.

**Chile. Metropolitana**: Río Clarillo National Reserve (Teillier et al. (2005).

#### Selected specimens examined.

**Chile. Metropolitana: [Santiago Province**] Valle de Mapocho, La Ermita, 33°20'S, 70°22'W, 2650 m a.s.l., 13 Mar 1992, *M.F. Gardner*, *A. Hoffmann & C. Page 5118* (E); La Parva, 33°19'48"S, 70°16'57"W, 2860 m a.s.l., 25 Jan 2008, *A. Moreira 1028* (SGO); [**Cordillera Province**] Tupungato, Río Colorado, Baños Salinillas, 1500 m a.s.l., 4 Jan 1930, *F. Behn s.n.* (CONC).

**11. *Schizanthus litoralis* Phil., Anal. Univ. Chile 91: 118. 1895**

### 11a. Schizanthus litoralis var. litoralis

Taxon classificationPlantaeSolanalesSolanaceae

1E65C7CE-3E99-5100-BECF-E0F5614BEAB5

Fig. 5A–D

#### Type.

Chile. Valparaíso: Médanos Concon, 10 Dec 1884, *F. Philippi s.n.* (lectotype designated by Grau and Gronbach 1984, pg. 136 [as type]: SGO! [SGO000004531 acc. #055374]).

**Figure 5.**
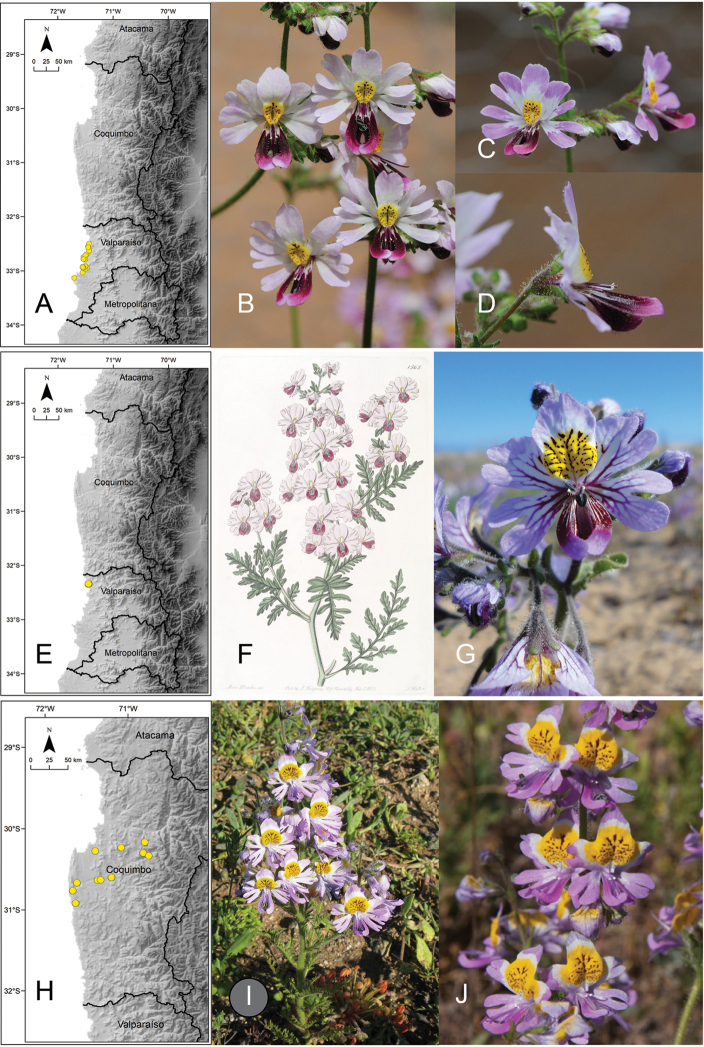
**A** distribution of Schizanthus litoralis var. litoralis**B–D** examples of S. litoralis var. litoralis in Colegio Sagrada Familia (Reñaca) **E** distribution of Schizanthus litoralis var. humilis**F** illustration of S. litoralis var. humilis published with the description of the species (Lindley 1833) **G** example of S. litoralis var. humilis in Pichicuy **H** distribution of *Schizanthus splendens***I** examples of *S. splendens* in Hacienda El Tangue (south of Tongoy) **J** road Ovalle-Socos. Photos by S. Elórtegui (**B–D**), V. Morales (**G**), A de Trenqualye (**I**), M. Aldunate (**J**).

#### Taxonomic notes.

For years, the name of this species was wrongly associated with the new species *S. carlomunozii* as growing from Coquimbo to Valparaíso (see notes under *S. carlomunozii*). The Fig. 5A–D depicts the true *S. litoralis* described by R.A. Philippi. In this case, the types of S. litoralis var. litoralis were especially important, as some flowers still show a darker colour on the lower lip, a character not mentioned by R.A. Philippi but that is distinctive of the species.

Most of the data on the lectotype matches other two specimens at SGO [SGO000004529 acc. #055325, SGO000004530 acc. #055326], except for the day and month of the collection (12^th^ October). However, none of them can be discarded as type material, because the original description only mentions the year of collection (1884). The similarities between day and month (10 = October and 12 = December), make us think that the date could be erroneously swapped on the labels. Unfortunately, we cannot tell with certainty which was the true date of collection or if these specimens correspond to the same collection. Therefore, we decided to treat these two specimens as syntypes, conserving the lectotype chosen by Grau and Gronbach (1984).

#### Key characters.

Delicate plant with flowers with noticeable dark purple to burgundy colour on the lower lip and faint or no spotting on any lobe, except on the yellow area of the upper middle lobe.

#### Distribution.

Endemic to Chile, in the coast of the Region of Valparaíso (between the Provinces of Petorca and Valparaíso, 32°30'–33°10' lat. S). Photos in Fig. 5B–D correspond to cultivated specimens, which were grown from seeds collected in Dunas de Concón, growing upon the paleodune. The original population at Dunas de Concón, apparently no longer exists due to urban development. 10–100 m a.s.l.

#### Habitat.

Schizanthus litoralis var. litoralis has been seen growing on sandy soil and between the fissures of the coastal rocks. Forms part of the coastal scrubland.

#### Conservation.

**Chile. Valparaíso**: Dunas de Concón Natural Sanctuary (apparently locally extinct).

#### Selected specimens examined.

**Chile. Valparaíso: [Valparaíso Province**] Dunas de Con Con, sobre las dunas a 100 m de la placa de Santuario mirando al mar, 32°56'36"S, 71°32'40"W, 30 m a.s.l., 7 Oct 2002, *A. Moreira 688* (SGO); Road along the coast, S. of Cachagua, N of Quintero, 32°36'35"S, 71°25'57"W, 100 m a.s.l., 10 Nov 2006, *E.J. Tepe*, *A. Marticorena & P.B. Pelser 1947* (CONC); N von Valparaíso, Oct 1967, *O. Zoellner 1856* (L).

### 11b. Schizanthus litoralis var. humilis

Taxon classificationPlantaeSolanalesSolanaceae

(Lindl.) V.Morales & Muñoz-Schick comb. nov.

F1FA07D0-A3A6-5087-AE6C-F70E82D0B166

urn:lsid:ipni.org:names:77210705-1

Fig. 5E–G

Schizanthus pinnatus var. humilis Lindl., Edwards’s Bot. Reg. 18: T. 1562. 1833.
Schizanthus tricolor Grau & E.Gronbach. Mitt. Bot. Staatssamml. München 20: 143. 1984.

#### Type.

Chile. Valparaíso: *H. Cuming 712* (lectotype designated by Grau and Gronbach 1984, pg. 143 [as type]: BM! [BM000994719]; isolectotypes: E! [E00089563, E00089564], MEL! [MEL-2449923 pro parte A]).

#### Taxonomic notes.

This taxon was first described by Lindley (1833). He recognised that the specimens available exhibited two well-defined characters; the flowers grouped in congested racemes and the total height of the plants lower than those mentioned for *S. pinnatus*. The second character was used by Lindley to describe this taxon as a dwarf variety of *S. pinnatus*.

Regarding the type material, it appears that Lindley (1833) had access to cultivated material as well as material from the wild. First, he cites living material that flowered in June of 1832 at the private garden of the Comte de Vandes, at Bayswater (London). In the same paragraph, the author establishes that the plants were the product of seeds collected by Hugh Cuming. What is not clear is if Lindley had access to fresh or dried samples from the cultivated plants. In the following paragraph, he mentions some dried specimens collected in Chile by Cuming under the number 712. Therefore, the group of samples seen by Lindley should be treated as syntypes, thus requiring necessary lectotypification. We have not found herbarium sheets that agree with the data from cultivated material. Instead, we have found four specimens numbered as 712 by Cuming that match the characters of the species. One of them has been selected as lectotype of Lindley’s name by Grau and Gronbach (1984: 143). This is a very poor sample of the species, that only shows the basal leaves of a single plant with a label saying “*Schizanthus*, *Cum 712*, *3898*”. Of the specimens at E, one is the best-preserved sample [E00089563] and the other shows a more detailed locality (Valparaiso) [E00089564]. Here we recognise all the specimens collected by Cuming 712 as a single collection and as isolectotypes.

#### Key characters.

It has a dark purple colour on the lower lip, but it differs from S. litoralis var. litoralis because of the distinct purple venation at the base of each corolla lobe.

#### Distribution.

Endemic to Chile, along the coast of the Region of Valparaíso (Province of Petorca, 32°20'–32°35' lat. S). This variety has been reported from the Dunes in Pichicuy and also in Cachagua (Villagrán et al. 2007). However, only the population in Pichicuy has been observed during the last decade

#### Habitat.

It grows within a narrow zone on the foredunes, associated with *Ambrosia chamissonis* Greene (Asteraceae), *Solanum coquimbense* J.R.Benn. (Solanaceae) and *Senecio bahioides* Hook. & Arn. (Asteraceae).

#### Conservation.

**Chile. Valparaíso**: Humedal de Pichicuy Protected Area.

#### Selected specimens examined.

**Chile. Valparaíso: [Petorca Province**] Final sur Playa Pichicuy, 32°20'24.1"S, 71°26'50.3"W, 10 m a.s.l., 12 Aug 2008, *M. Muñoz 5000* (SGO); Pichicuy, dunes behind beach, 32°20'35"S, 71°27'05"W, 2 m a.s.l., 9 Nov 2006, *E.J. Tepe*, *A. Marticorena & P.B. Pelser 1886* (CONC); Zapallar (médanos de Cachagua), 25 Sep 1909, *F. Johow s.n.* (CONC).

### 12. Schizanthus splendens

Taxon classificationPlantaeSolanalesSolanaceae

Sudzuki, Agricultura Técnica, Chile 5(1): 33. 1945

584996AF-017B-5A8E-A39E-660ADFA2DE87

Fig. 5H–J

#### Type.

Chile. Coquimbo: Llano de Los Loros, 10 Sep 1942, C. Muñoz & E. Pisano 3349 (lectotype here designated: SGO! [SGO000004537 acc. #148994]; isolectotype: SGO! [SGO000004538 acc. #143619]).

#### Taxonomic notes.

In the protologue, the author mentions the holotype as being held at the National Museum of Natural History, Santiago. Within the type collection at SGO, we have found two sheets of this species, which are duplicates. In comparing the label data with the protologue, we identified some differences. In the description, the author cited Muñoz & Johnson as collectors of the specimens, but without giving a date of collection. This was an oversight because the herbarium specimens were collected by Carlos Muñoz & Edmundo Pisano and they clearly show the date of collection. This oversight could have occurred because Sudzuki had access to the specimens before they were mounted, in order that she could complete her thesis on the genus *Schizanthus*.

Grau and Gronbach (1984) did not recognise this name as an accepted species, citing it as synonym of *S. litoralis*. On page 136, the authors cited the type of *S. splendens* just giving the data from the protologue and without indicating the specimens as occurring at SGO. We are selecting as lectotype the specimen which has a label with the name of the species and the descriptor (“*S. splendens nov. sp. Sudzuki*”), while the second specimen was lacking this information.

#### Key characters.

Flowers similar in form to S. carlomunozii var. carlomunozii as they exhibit a white halo around the yellow area but without any dark spots outside of it. The flowers are subsessile with peduncles up to 4 mm long, instead of 0.5–2.5 cm in *S. carlomunozii*.

#### Distribution.

Endemic to Chile, in the Region of Coquimbo (Provinces of Elqui and Limarí, 30°10'–31°0' lat. S). 350–1800 m a.s.l.

#### Habitat.

Along roadsides and in flat areas; over clayed soil.

#### Conservation.

**Chile. Coquimbo**: Fray Jorge National Park.

#### Selected specimens examined.

**Chile. Coquimbo: [Elqui Province**] Cordillera de Ovalle, Cerro Tololo, 1800 m a.s.l., 26 Oct 1971, *C. Jiles 6302* (CONC); Andacollo 5 km al sur, 33°15'S, 71°06'W, 1100–1200 m a.s.l., 1–6 Apr 2008, *M. Mihoc 325* (CONC); [**Limarí Province**] Salida S de Ovalle, planicies, 22 Aug 1991, *M. Muñoz 2566* (SGO); Carretera Panamericana, 19 km al norte de la Quebrada del Teniente, 16 Oct 1971, *C. Marticorena*, *R. Rodríguez & E. Weldt 1440* (CONC, F).

**13. *Schizanthus porrigens* Graham ex Hook., Exot. Fl. 2: tab. 86. 1824**

### 13a. Schizanthus porrigens subsp. porrigens

Taxon classificationPlantaeSolanalesSolanaceae

31D5FBF5-9724-5C74-81CB-5CB2231B3445

Fig. 6A–E

Schizanthus porrigens Graham, Edinb. N. Phil. Journ. 11: 401. 1824.
Schizanthus tenuifolius Phil., Anal. Univ. Chile 91: 118. 1895.
Schizanthus tenuis Phil., Anal. Univ. Chile 91: 124. 1895.
Schizanthus heterophyllus Phil., Anal. Univ. Chile 91: 125. 1895.

#### Type.

United Kingdom. Scotland: Hort. Edin. [cultivated material at the Royal Botanic Garden Edinburgh] (lectotype designated here: E! [E00089607]; isolectotype: E! E00089608]).

**Figure 6.**
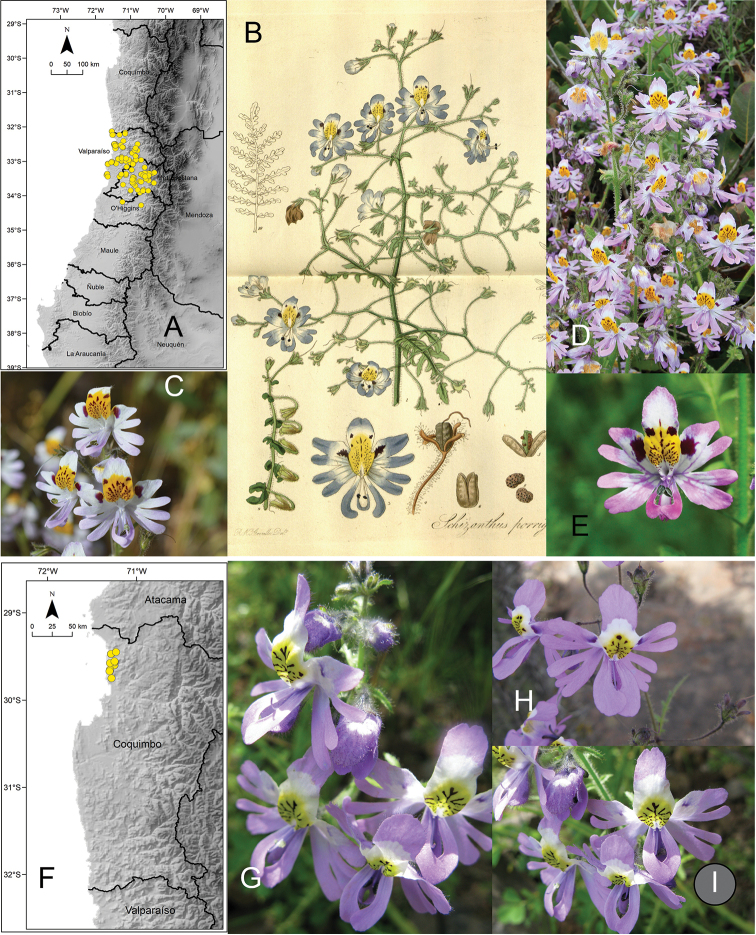
**A** distribution of *Schizanthus porrigens***B** illustration of *S. porrigens* published with the description of the species (Hooker 1824) **C** examples of *S. porrigens* in Putaendo **D** Cerro La Huinca (Limache) **E** Los Molles (Valparaíso Region) **F** distribution of Schizanthus porrigens subsp. borealis**G–I** examples of S. porrigens subsp. borealis in Cuesta Buenos Aires (north of La Serena). Photos by A. Cádiz (**C**), A. Moreira-Muñoz (**D, E, G–I**).

#### Taxonomic notes.

The name *Schizanthus porrigens* was first used by Graham (1824) when he listed this rare plant growing outside at the Royal Botanic Garden Edinburgh. Grau and Gronbach (1984: 134) accepted this name as being validly published by Graham, but in our opinion, Graham’s (1824) is not a valid publication of the name, as it only provides a short comparison to *S. pinnatus*, based on the smaller first emerging leaves (“*This species may be distinguished from S. pinnatus even in the seed bed, but the seminal leaves being shorter.*”). Hence, the specimen cited by Grau and Gronbach (“*s.n. Graham* (*E*)”), should be not considered as type of the name validly published by W.J. Hooker and illustrated by R.K. Greville.

Studying the original description, it is clear to us that the species was described based on cultivated material. Hooker (1824) stated that the plants at the Royal Botanic Garden Edinburgh were growing with individuals of another species, which was previously published by him (Hooker 1823) (as *S. pinnatus*, but represents S. litoralis var. litoralis). Hooker (1823) stated that the seeds were collected by Mr. Cruikshanks [Cruckshanks] and sent to Dr. Graham in Edinburgh. Hooker (1824) cited much information from Graham in his description of *S. porrigens*.

In trying to locate the type material, we searched for specimens at E and K, as these are the herbaria which hold most of the type specimens by W.J. Hooker (see Taxonomic notes under *S. grahamii*). At K, we could not find any specimens of *S. porrigens* but we found 15 samples at E (https://data.rbge.org.uk/search/herbarium/). Of these, only five were labelled as *S. porrigens* [E00089607, E00089608, E00089609, E00089610, E00089611]. The first two sheets were identified as possible types of the name *S. porrigens* Graham by Gronbach in 1983. We think these two specimens are part of the same collection, as both have Greville’s handwriting and show complementary parts of an individual plant: the specimen E00089607 shows the inflorescence while E00089608 comprises a branch with several leaves. We think these specimens could have been used by Greville for illustrating the description. On the other hand, sheets E00089609, E00089610, E00089611 originated from GL and have the handwriting of W.J. Hooker (H. Noltie 2019, pers. comm.). Additionally, we found two herbarium sheets at P that can be related to the description [P00477566, P00477611]. The first one says “*Schizanthus porrigens Hook. Fl. Exot. E Chile* (*misit Hooker 1824*) *4733*”. The second sheet has the same information, except for the name of the species “*Schizanthus pinnatus*”. Both labels were handwritten by Hooker, except for the annotation “*misit Hooker 1824*”, which could have been added by E. Drake, former owner of the collection, meaning that the specimens were sent to him by Hooker in 1824 (H. Noltie 2019, pers. comm.). This data matches the year when Hooker described the species. Surely the specimens are duplicates and were part of an exchange of material between herbaria from the United Kingdom and France. We think specimens listed above should be treated as original material of the name *Schizanthus porrigens* Graham ex Hook. Following Turland et al. (2018: Art. 9.3.), we select the sheet E00089607 as lectotype and E00089608 as isolectotype, as they are considered duplicates and the only sheets that we can clearly relate to the description by Hooker (1824), even though it seems that all other specimens were seen by him.

#### Key characters.

Schizanthus porrigens subsp. porrigens is a very variable species, especially in the colour of the flowers, which vary from intense rose and sometimes bluish to white. The spots are also variable; with two little ones at the sides of the upper middle lobe and two slightly larger at the upper lateral lobes (in the separation of the lateral and middle lobes). Sometimes, it has a purple irregular line above the yellow area. When the corolla is mostly white, the lower lip has a larger portion coloured light purple, but the same colour also occurs at the margin of the upper lateral lobes.

#### Distribution.

Endemic to Chile, between the Regions of Coquimbo (Province of Choapa, 32°5' lat. S) and O’Higgins (Province of Cachapoal, 34°20' lat. S). 20–1800 m a.s.l.

#### Habitat.

One of the most widespread species inhabiting from the coast to the cordillera, in different types of substrates; in shrubby and sclerophyllous communities.

#### Protected areas.

**Chile. Valparaíso**: La Campana National Park, BioParque Puquén-Los Molles Private Reserve, Acantilados Federico Santa María Natural Sanctuary, Palmar El Salto Natural Sanctuary, Serranía El Ciprés Natural Sanctuary; **Metropolitana**: Río Clarillo National Reserve, Cerro El Roble Natural Sanctuary, Yerba Loca Natural Sanctuary, Altos de Cantillana Natural Sanctuary.

#### Selected specimens examined.

**Chile. Coquimbo: [Choapa Province**] Pichidangui, 32°9'S, 71°31'W, 15 m a.s.l., 18 Nov 1960, *J. Petersen s.n.* (CONC); **Valparaíso: [Petorca Province**] 3 km al S de Papudo, 32°31'S, 71°28'W, 50 m a.s.l., 10 Nov 2001, *C. Aedo 6817* (CONC); Caleta Los Molles, inicio sendero al Puquén, 32°14'11.3"S, 71°30'59.7"W, 217 m a.s.l., 19 Oct 2008, *M. Muñoz 5070* (SGO); [**Quillota Province**] Cuesta El Melón, 32°40'S, 71°15'W, 500 m a.s.l., 10 Feb 1990, *C. von Bohlen 650* (CONC); Parque Nacional La Campana, sector Ocoa, sobre la cascada, 32°57'40.428"S, 71°3'13.86"W, 10 Dec 2000, *A. Moreira 487* (SGO); [**San Felipe Province**] Cuesta Las Chilcas, 32°51'1.5"S, 70°51'32.8"W, 508 m a.s.l., 19 Oct 2008, *M. Muñoz 5065* (SGO); Santuario Serranía El Ciprés, 32°41'12"S, 70°48'14"W, 1167 m a.s.l., 5 Oct 2013, *A. Madrid & J. Larraín 79* (CONC); [**Valparaíso Province**] Viña del Mar (El Salto) langs de weg, 11 Nov 1937, *C. Andreas 24* (L, U); Quilpué, Teniente Serrano – Poza Larga, coord. UTM 274802E – 6337967W, 19 Nov 2004, *O. Fernández 1092* (CONC); Cuesta Zapata, 300 m a.s.l., 27 Oct 1990, *C. von Bohlen 834* (SGO); [**San Antonio Province**] El Quisco, Nov 1976, *H. Gunckel & H. Vergara s.n.* (CONC); El Tabo, Quebrada de Córdoba, 33°25'S, 7 Oct 1980, *C. Villagrán 2878* (SGO); **Metropolitana: [Chacabuco Province**] Altos de Chicauma, sector Loma Blanca, 33°12'S, 70°56'W, 750 m a.s.l., 29 Sep 2002, *N. García*, *C. Valdivia & F. Salinas 3294* (CONC); Montenegro, UTM 19H 0327491-6351928, 12 Oct 2002, *M. Muñoz 4155* (SGO); [**Santiago Province**] Santuario de la Naturaleza Yerba Loca, laderas al borde del estero de la Yerba Loca, cerca de la confluencia con el río San Francisco, 33°19'S, 70°19'W, 1800 m a.s.l., 16 Dec 1999, *M.T.K. Arroyo*, *C. Valdivia & P. McPherson 994740* (CONC, SGO); [**Cordillera Province**] San José de Maipo, 12 Oct 1969, *A. Cid 13* (CONC); La Obra, 820 m a.s.l., 20 Nov 1927, *G. Montero 481* (F); [**Maipo Province**] Cerro Lo Chena, 780 m a.s.l., 26 Nov 1950, *H. Gunckel s.n.* (CONC); Fundo Cullipeumo, Cerro Cullipeumo, Champa, 21 Nov 1976, *I. Gallardo s.n.* (SGO); [**Melipilla Province**] Camino entre Chorombo y Casablanca, en la cuesta, 33°27'S, 71°19'W, 280 m a.s.l., 17 Sep 2009, *I. Escobar 202* (CONC); Alhué, lado del río, 18 Nov 2002, *A. Brinck s.n.* (SGO); Cuesta de Barriga, between Marruecos and Los Cerrillos, 850 m a.s.l., 3 Nov 1948, *E.P. Killip & E. Pisano 39675* (US); [**Talagante Province**] Talagante, Sep 1969, J Salas s.n. (CONC); Mallarauco, 31 Oct 1988, *J.P. Schiappacase s.n.* (F); **O’Higgins: [Cachapoal Province**] Palmería Cocalán, 2 Sep 2004, *Fundación Philippi 89* (SGO); Angostura de Paine, 25 Oct 1969, *O. Zoellner 3461* (CONC); Camino de Rancagua a Caletones, km 14, 1000 m a.s.l., 17 Nov 1970, *C. Marticorena & E Weldt 639* (CONC).

### 13b. Schizanthus porrigens subsp. borealis

Taxon classificationPlantaeSolanalesSolanaceae

V.Morales & Muñoz-Schick subsp. nov.

FC868D4B-8C4B-51B7-A04C-723AFD89B62E

urn:lsid:ipni.org:names:77210706-1

Fig. 6F–I

#### Diagnosis.

Differs from subsp. porrigens in its uniform corolla colour, with or without one faint dark spot in the upper part of each lateral lobe (in the separation of the lateral and middle lobe). These spots do not reach the margin of the lobes. A white halo always surrounding the yellow area of the upper middle lobe.

#### Type.

Chile. Coquimbo: norte La Serena-Cuesta Porotitos, N El Arrayán, 29°40'10.5"S, 71°18'17.6"W, 184 m alt., 7 Dec 2008, *M. Muñoz 5023* (holotype: SGO! [acc. #157830]).

#### Description.

Annual plant, glandular-pilose, with one or several stems arising from the same root, up to 50 cm tall. Leaves bipinnatifid, the blade 5.5–7.5(9) cm long, 1.5–2(2.5) cm wide; segments irregularly divided (opposite or alternate), diagonal or perpendicular to the midrib. Inflorescence 9–24 cm long, with basal peduncles up to 10–25 mm long and apical peduncles 4–7 mm long. Calyx hirsute-glandular, the divisions linear-spathulate of 4–6 mm long, 1–1.5 mm wide. Corolla with the tube shorter than the calyx, up to 3 mm long; limb bluish to lilac, 22–28 mm long, 15–20 mm wide; upper middle lobe 12–16 mm long, 5–6 mm wide, oblanceolate, the apex obtuse or sometimes a little retuse, little dotted with dark spots over the yellow area and surrounded by a white halo; upper lateral lobes without spots or sometimes with a small spot in each side (in the separation of the lateral and middle lobes), these spots not reaching the upper margin of each lobe; lower middle lobe 7–9 mm long, 4–6 mm wide; lateral lobes 10–13 mm long, 2–3.5 mm wide, linear-spathulate, longer than the middle lobe. Stamens reaching half of the length of the lower middle lobe. Capsule as long as the calyx, glabrous.

#### Taxonomic notes.

Grau and Gronbach (1984) considered this new taxon as part of the variability of *S. porrigens*. This can be seen in their drawings (Grau and Gronbach 1984: Abb. 22, 23), based on specimens collected between Playa Temblador and Cruz Grande, in the Coquimbo region.

We have named this taxon as subsp. borealis, meaning its populations have a more northern distribution than the typical subspecies.

#### Key characters.

Schizanthus porrigens subsp. borealis has a bluish or lilac uniform corolla colour, without or with one faint dark spot in the upper part of each lateral lobe (in the separation of the lateral and middle lobe). These spots do not reach the margin of the lobes.

#### Distribution.

Endemic to Chile, in the coast of the Region of Coquimbo (Province of Elqui, 29°25'–29°50' lat. S). 170–600 m a.s.l.

#### Habitat.

Inhabits shady hillsides and ravines, rocky outcrops with large boulders. It can be found among dense vegetation dominated by *Heliotropium stenophyllum* Hook. & Arn. (Heliotropiaceae), Senna cumingii var. coquimbensis (Vogel) H.S.Irwin & Barneby (Fabaceae), *Ophryosporus triangularis* Meyen (Asteraceae) and *Lobelia polyphylla* Hook. & Arn. (Campanulaceae). Also present in grazed areas with a dominance of shrubby species of Asteraceae.

#### Conservation.

**Chile. Coquimbo**: Santa Gracia Private Natural Reserve (N. Mercado, https://www.inaturalist.org/observations/8988561).

#### Specimens examined.

**Chile. Coquimbo: [Elqui Province**] Road between La Serena and Vallenar, 4 Oct 1971, *K. Beckett*, *M. Cheese & J. Watson 4060* (SGO); About 45 km N of La Serena along coast and ca. 10 km NW of the Panamerican (hwy 5) on road to Totoralillo, 21 Sep 1991, *L.R. Landrum & S.S. Landrum 7514* (ASU, CONC); Env. 30 km au N de La Serena, montée vers mine El Tofo, 29°35'S, 71°15'W, 500 m a.s.l., 23 Oct 1991, *F. Billiet & B. Jadin 5320* (US); La Serena, 4,6 kms from Chungungo en route to El Temblador, 29°28'18.6"S, 71°17'19.8"W, 171 m a.s.l., 30 Nov 2004, *P. Baxter*, *M.F. Gardner*, *P. Hechenleitner*, *P.I. Thomas & E. Zamorano 1765* (E, SGO); Primera curva de bajada Cuesta Buenos Aires, 29°33'20.2"S, 71°14'53.6"W, 566 m a.s.l., 8 Oct 2008, *M. Muñoz 5053* (SGO); Cuesta de Buenos Aires, cerca del portezuelo, 550 m a.s.l., 20 Oct 1971, *C. Marticorena*, *R. Rodríguez & E. Weldt 1586* (CONC, F); Al pié de la Cuesta de Buenos Aires, 18 Sep 1958, *E. Bailey s.n.* (CONC, SGO); Cuesta Buenos Aires, 29°34'S, 71°14'W, 500 m a.s.l., 31 Oct 1991, *R. Rodríguez 2786* (CONC); Subida S Cuesta Buenos Aires, 29°34'S, 71°20'W, 27 Oct 1991, *M. Muñoz*, *S. Teillier*, *I. Meza 2639* (SGO); Cuesta Buenos Aires a Playa Temblador, primeros kilómetros, 28 Sep 1997, *M. Muñoz 3808* (SGO); Quebrada Honda, 41 km N La Serena, 1km del mar, 40 m a.s.l., 26 Sep 1948, *R. Wagenknecht 323* (CONC); Quebrada Honda, falda occidental del Cerro Juan Soldado, 200–300 m a.s.l., 4 Nov 1949, *W. Biese* 3021 (SGO); Panamericana Norte, entre La Serena y Caleta Hornos, antes Puente Juan Soldado, 3 Oct 1991, *C. von Bohlen 1199* (SGO); *C. von Bohlen 1191* (SGO); Km 495 al norte Cuesta Porotitos, 11 Oct 1992, *M. Muñoz 3029* (SGO).

### 14. Schizanthus carlomunozii

Taxon classificationPlantaeSolanalesSolanaceae

V.Morales & Muñoz-Schick sp. nov.

F0141176-B0D3-5920-891C-A6BEF063A1CE

urn:lsid:ipni.org:names:77210707-1

#### Diagnosis.

Differs from all other species in the genus in its large flowers with a very distinctive pattern on the upper lip of the corolla, where distinct spots cover the margins of the middle and lateral lobes.

#### Type.

Chile. Coquimbo: Carretera Panamericana, entre la Quebrada de El Teniente a Talinay, 21 Aug 1963, *C. Muñoz & E. Sierra s.n.* (holotype: SGO! [acc. #075670]; isotype: SGO! [acc. #075671]).

### 14a. Schizanthus carlomunozii var. carlomunozii

Taxon classificationPlantaeSolanalesSolanaceae

V.Morales & Muñoz-Schick

CD2CF3AE-790A-5851-A0F0-5C3BCCFC0870

Fig. 7A–D

#### Description.

Annual plant, glandular-pilose, with one or several stems arising from the same root, up to 45 cm tall. Leaves bipinnatifid, the blade 6.5–9 cm long, 2–3.5 cm wide; segments irregularly divided (opposite or alternate) and perpendicular to the midrib. Inflorescence 15–28 cm long, with basal peduncles up to 30–40 mm long and apical peduncles 4–8 mm long. Calyx hirsute-glandular, the divisions 5–8 mm long, 1.5–3 mm wide, linear-spathulate. Corolla with the tube shorter than the calyx, up to 3 mm long; limb pale violet, sometimes whitish, 20–28(34) mm long, 18–30(40) mm wide; upper lip with distinct dark spots on the margins of the middle lobe and in the upper part of the lateral lobes (in the separation of the lateral and middle lobes); upper middle lobe 10–18 mm long, 7–9(12) mm wide, oblanceolate, the apex obtuse to retuse, dotted with dark spots over the yellow area, sometimes surrounded by a white halo; upper lateral lobes with the upper segments a little rounded downwards and wider than the lower segments; lower middle lobe 7–8 mm long by 4–6 mm wide; lateral lobes 10–12(17) mm long, 2(4) mm wide, linear-spathulate, longer than the middle lobe. Stamens almost reaching the length of the lower middle lobe. Capsule as long or little longer than the calyx, glabrous.

**Figure 7.**
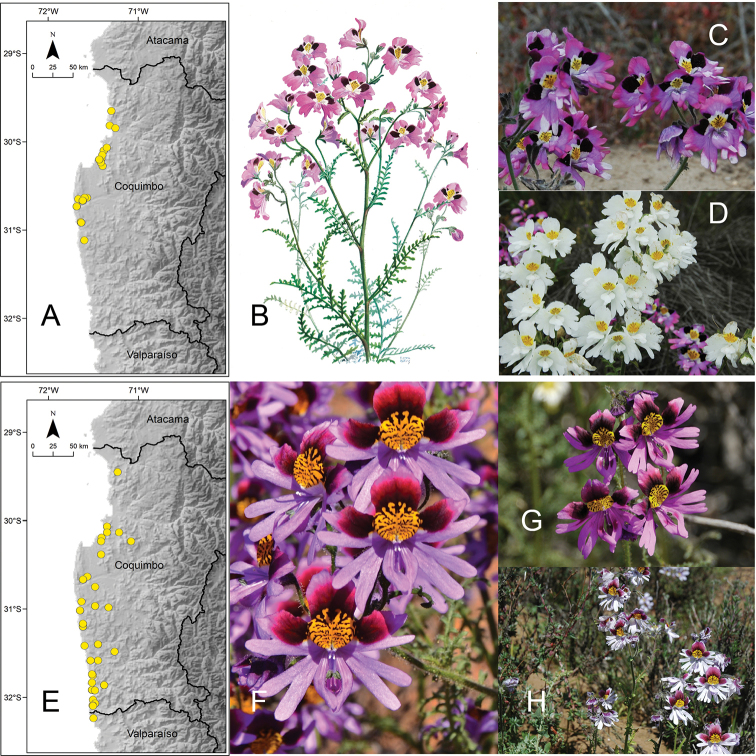
**A** distribution of Schizanthus carlomunozii var. carlomunozii**B** illustration of S. carlomunozii var. carlomunozii by S. Rafols (Muñoz-Pizarro 1966) **C, D** examples of S. carlomunozii var. carlomunozii in Fray Jorge National Park **E** distribution of Schizanthus carlomunozii var. dilutimaculatus**F** examples of *S. carlomunozii var. dilutimaculatus* in Amolanas **G** Las Majadas **H** El Ñague. Photos by M.T. Eyzaguirre (**C, D, G, H**), M. Aldunate (**F**).

#### Taxonomic notes.

Grau and Gronbach (1984), following Reiche (1909: 477), treated this species as part of their concept of *S. litoralis*. We consider their description and figures include two different taxa and none of them correspond to the true *S. litoralis*; the abb. 24–27 in Grau and Gronbach (1984), shows the variation which corresponds to *S. carlomunozii*. All these drawings were based on samples collected in the Region of Coquimbo. This area corresponds to the distribution of this new species.

The name of the species honours the highly regarded Chilean botanist Carlos Muñoz Pizarro, for he provided a complete description that includes an illustration of this taxon in “*Flores silvestres de Chile*” (Muñoz-Pizarro 1966), but erroneously naming it as *S. litoralis*. Variability of the corolla drawings leads us to recognise two varieties within the species; one with two distinct spots on the margins of the upper middle lobe, and another with a large spot of fading colour of the same width of the upper middle lobe, which we described as var. dilutimaculatus.

#### Key characters.

Schizanthus carlomunozii var. carlomunozii differs from all other species in the genus in its large flowers (20–34 mm long, 18–40 mm wide) with a very distinctive pattern on the upper lip of the corolla, with one medium or large delimited spot in the upper part of each lateral lobe (in the separation of the lateral and middle lobes). These spots reaching the margin of the lobes. The upper middle lobe also with two small or medium dark spots on the margins of it.

#### Distribution.

Endemic to Chile, along the coast of the Region of Coquimbo (Provinces of Elqui and Limarí, 29°35'–31°10' lat. S). 20–350 m a.s.l.

#### Habitat.

It grows under the shade of shrubs close to the sea; and along roadsides, sandy slopes and dry fields.

#### Conservation.

**Chile. Coquimbo**: Fray Jorge National Park.

#### Specimens examined.

**Chile. Coquimbo: [Elqui Province**] Fundo Juan Soldado, 7 km al norte de La Serena, 1 Oct 1941, *R. Wagenknecht s.n.* (CONC); La Serena (Punta Teatinos), Sep 1898, *K. Reiche s.n.* (SGO); Norte de La Serena, frente Transportes Depetris, en arenales cerca línea de tren, 29°50'39.4"S, 71°15'16.6"W, 18 m a.s.l., 8 Oct 2008, *M. Muñoz 5057* (SGO); La Serena, Sep 1926, *E. Barros 2373* (CONC); Las Tacas, 8 Oct 2000, *A. Brinck s.n.* (SGO); Carretera entre Guanaqueros y La Serena, 18 Sep 1983, *M. Muñoz 1828* (SGO); Quebrada Tongoycillo, 19 Sep 1948, *C. Jiles 859* (CONC); Depto. Ovalle, Guanaqueros, 13 Sep 1965, *F. Behn s.n.* (CONC); Guanaqueros, 30 m a.s.l., Sep 1965, *C. Muñoz 5* (SGO); Camino interior Guanaqueros a Tongoy, primer km, 12 Oct 1989, *M. Muñoz 2418* (SGO); Dpto. Ovalle, Carretera Panamericana, frente a Tongoy, 30°15'S, 71°30'W, 30 m a.s.l., 20 Sep 1961, *F. Schlegel 3922* (CONC); [**Limarí Province**] Carretera Panamericana, 7 km al norte de la Quebrada Los Almendros, 17 Oct 1971, *C. Marticorena*, *R. Rodríguez & E. Weldt 1468* (CONC, LP); Lagunillas, 21 Sep 1972, O. Zoellner 6198 (L); Prov. Elqui, Lagunillas, 30°6'S, 71°21'W, 100 m a.s.l., Sep 1987, *F. Squeo 87067* (CONC); Idem, *F. Squeo 87033* (CONC); Camino acceso Parque Nacional Fray Jorge, 30°38'1.4"S, 71°26'18"W, 305 m a.s.l., 11 Oct 2004, *M. Muñoz 4484* (SGO); Fundo Las Garzas, entre carretera Panamericana y el Bosque Fray Jorge, a 5 km al poniente, 14 Sep 1957, *C. Muñoz 4284* (SGO); Parque Nacional Fray Jorge, road between information center/gift shop and the Sendero interpretativo del Bosque Hidrófilo, 30°38'53"S, 71°40'0"W, 251 m a.s.l., 4 Nov 2006, *E.J. Tepe*, *A. Marticorena & P.B. Pelser 1724* (CONC); Dpto. Ovalle, Fray Jorge, parte baja, 20 Sep 1952, *M. Ricardi 2086* (CONC); Idem, *M. Ricardi 2095* (CONC); Fray Jorge, 18 Sep 1970, O. Zoellner 4388 (L); Estancia Frai Jorge, 215 m a.s.l., 13 Aug 1917, C. Skottsberg 742 (F); Fundo Fray Jorge, 100 m a.s.l., Sep 1934, *C. Grandjot & G. Grandjot 466a* (CONC); Dpto. Ovalle, Fray Jorge, cerca de las casas, 30°40'S, 71°40'W, 180 m a.s.l., 22 Aug 1948, *C. Jiles 751* (CONC); Dpto. Ovalle, Fray Jorge, 30°40'S, 71°40'W, 350 m a.s.l., Sep 1958, *J. Kummerow s.n.* (CONC); Dpto. Ovalle, Fray Jorge, 30°40'S, 71°40'W, 350 m a.s.l., 13 Sep 1961, *B. Behn s.n.* (CONC); Dep. Ovalle, Fray Jorge, ca. 300 m a.s.l., Nov 1925, *E. Werdermann 912* (E, F, SI, U, US); Monte de Fray Jorge, Sep 1904, *K. Reiche s.n.* (SGO); Fray Jorge, 26 Sep 1935, *C. Muñoz 268* (SGO); *C Muñoz 240* (SGO); *C. Muñoz 131* (SGO); Fray Jorge, 30 Oct 1956, *M. San Martín 650* (SGO); Depto. Ovalle, Fray Jorge, 8 Oct 1947, *B. Sparre 2903* (SGO); Ovalle, Fray Jorge, 15 Sep 1947, *Ibáñez & Kuschel s.n.* (SGO); Desembocadura Río Limarí, 12 Sep 1942, *C. Muñoz 3417* (SGO); Dpto. Ovalle, Estancia Talca, 30°54'S, 71°39'W, 300 m a.s.l., 19 Sep 1949, *C. Jiles 1435* (CONC); Faldeos del Cerro Talinay, 15 Sep 1957, *M. Ricardi & C. Marticorena 4284/669* (CONC); 122 km al N de Los Vilos, 19 Sep 1991, *C. Fernández & H. Niemeyer* (*91*)*183* (SGO); Más o menos al Sur de Mantos de Hornillos, alrededor del km 290, 21 Sep 1986, *M. Muñoz 2072* (SGO); **Unknown**: Coquimbo, La Rinconada, 17 Sep 1952, *E. Barros 10126* (CONC); Litoral de Coquimbo, Sep 1898, *K. Reiche s.n.* (SGO); Chili, 1828/1834, *C. Gay 176* (P).

### 14b. Schizanthus carlomunozii var. dilutimaculatus

Taxon classificationPlantaeSolanalesSolanaceae

V.Morales & Muñoz-Schick var. nov.

83893DE6-B6A3-577A-80C9-1ACD8C544835

urn:lsid:ipni.org:names:77210708-1

Fig. 7E–H

#### Diagnosis.

Similar to S. carlomunozii var. carlomunozii but differing in possessing three large and dark spots covering the distal portion of the upper lip. The colour of these spots fades from the bottom to the top.

#### Type.

Chile. Coquimbo: 1 km S Los Vilos, 31°55'17"S, 71°29'03"W, 6 Oct 2008, *M. Muñoz 5020* (holotype: SGO! [acc. #157833]).

#### Description.

Annual plant, glandular-pilose, with one or several stems arising from the same root, up to 70 cm tall. Leaves bipinnatifid, the blade 4.5–7.5(13) cm long, 1.5–3.5(4) cm wide; segments irregularly divided (opposite or alternate) and perpendicular to the midrib. Inflorescence 5–30 cm long, with basal peduncles up to 30–40 mm long and apical peduncles up to 4–14 mm long. Calyx hirsute-glandular, the divisions linear-spathulate of 5–9(12) mm long, 1–2 mm wide. Corolla with the tube shorter than the calyx, up to 3 mm long; limb pink, lavender or lilac, sometimes whitish, 20–28 mm long, 15–25 mm wide; upper lip with three large spots of colour black to burgundy, which fade from the bottom to top. One of these spots occupies the total width of the upper half of the middle lobe, while the others two occupy the upper part of the lateral lobes; upper middle lobe 12–15 mm long, 7–9 mm wide, oblanceolate, the apex obtuse to retuse, dotted with dark spots over the yellow area; upper lateral lobes with the upper segments a little rounded downwards and wider than the lower segments; lower middle lobe 6–9 mm long, 4–6 mm wide; lateral lobes 10–12 mm long, 2 mm wide, linear-spathulate, longer than the middle lobe. Stamens almost reaching the length of the lower middle lobe. Capsule shorter or little longer than the calyx, glabrous.

#### Taxonomic notes.

The name selected to this variety refers to the coloured dark spots, which fade from the bottom to the top and cover the distal portion of the upper lip.

#### Key characters.

Similar to S. carlomunozii var. carlomunozii in the size and corolla form but differing in possessing three large and dark spots covering the distal portion of the upper lip. The colour of these spots fade from the bottom to the top.

#### Distribution.

Endemic to Chile, occurs along the coast between the Regions of Coquimbo (Province of Elqui, 29°25' lat. S) and Valparaíso (Province of Petorca, 32°20' lat. S). 20–350 m a.s.l.

#### Habitat.

On dunes and in sandy soils, shady slopes and recently disturbed roadside verges. It grows under the shade of shrubs and is associated with *Centaurea chilensis* Bertero ex W.Bull (Asteraceae), *Myrcianthes coquimbensis* (Barnéoud) Landrum & Grifo (Myrtaceae) and *Heliotropium stenophyllum* Hook. & Arn. (Heliotropiaceae).

#### Conservation.

**Chile. Coquimbo**: Fray Jorge National Park.

#### Specimens examined.

**Chile. Coquimbo: [Elqui Province**] Panamericana, frente al Tofo, 9 Oct 1971, *E. Kausel 5458* (SGO); m/m 40 kms al sur de La Serena, 15 Sep 1957, *M. Ricardi & C. Marticorena 4330/715* (CONC); Carretera Panamericana, 40 km al S de La Serena, 15 Sep 1957, *A.L. Cabrera 12587* (LP, US); 20 km norte de Guanaqueros, lado carretera, ladera exp. Oeste, 11 Oct 1987, *C. von Bohlen 483* (SGO); Camino entre Guanaqueros y Coquimbo, 19 Sep 1980, *M. Muñoz 1662* (SGO); Entre Las Tacas hacia Totoralillo, al Sur de Coquimbo por la carretera panamericana,15 Sep 1957, *C. Muñoz 4168* (SGO); Idem, *C. Muñoz 4174* (SGO); Depto. Ovalle, Quebrada Tongoicillo, 30°10'S, 71°21'W, 250 m a.s.l., 19 Sep 1948, *C. Jiles 850* (CONC); Andacollo, Estación H/S Acueducto Andacollo, 750 m a.s.l., 12 Oct 1971, *A. Flores & M. Flores 24* (SGO); A 5 km de El Peñón camino hacia Andacollo, 30°12'S, 71°9'W, 500–600 m a.s.l., 1–6 Apr 2008, *M. Mihoc 438* (CONC); Camino Guanaqueros – Tongoy, 27 Sep 1997, *M. Muñoz 3801* (SGO); Depto. Ovalle, Estancia Camarones, 30°20'S, 71°25'W, 75 m a.s.l., 19 Oct 1961, *C. Jiles 3855* (CONC); [**Limarí Province**] Entre Panamericana y Fray Jorge (Coquimbo), 19 Oct 1963, *A. Garaventa s.n.* (CONC); Bosque Fray Jorge, Sep 1971, *O. Muñoz s.n.* (CONC); Parque Nacional Fray Jorge, 18 Oct 2000, *N. Muñoz s.n.* (SGO); Sur de Socos, 22 Sep 1991, *I. Moreira 4* (SGO); Hacienda Talinay (Prov. de Coquimbo), 12 Oct 1961, *A. Garaventa s.n.* (CONC); Ovalle, El Parral, 500 m a.s.l., 4 Sep 1950, *C. Jiles 1853* (CONC); Dpto. Ovalle, Las Tunas, 11 Sep 1952, *C. Jiles 2152* (CONC); Dto. Ovalle, Quebrada Teniente, 19 Sep 1952, *M. Ricardi 2048* (CONC); Al norte de Mantos de Hornillos, 1 km antes de la Quebrada del Teniente, 13 Oct 1963, *C. Marticorena & O. Matthei 138* (CONC); Sur Bahía El Teniente, 270 m a.s.l., 22 Sep 1991, *M. Muñoz 2577* (SGO); Corral de Julio, exclusión La Rojadilla, 150 m a.s.l., 6 Nov 1976, *M. Muñoz 919* (F, SGO); Dpto. Ovalle, Quebrada Amolanas, 31°12'S, 71°36'W, 260 m a.s.l., 3 Oct 1948, *C. Jiles 985* (CONC); Idem, *C. Jiles 921* (CONC); [**Choapa Province**] Dpto. Illapel, Caleta Oscuro, 31°25'S, 71°35'W, 5–50 m a.s.l., 2 Nov 1974, *C. Marticorena*, *O. Matthei & R. Rodríguez 326* (CONC); Canela Baja, 11 Sep 1997, *O. Zoellner 21985* (CONC); Canela Baja a 11 km de la Carretera, 310 m a.s.l., 5 Oct 1997, *M. Muñoz 3846* (SGO); Salida camino Canela a Ruta 5 sur, 5 Oct 1997, *M. Muñoz 3850* (SGO); Dpto. Illapel, Carretera Panamericana, 10 km al norte de Huentelauquén, 16 Oct 1971, *C. Marticorena*, *R. Rodríguez & E. Weldt 1417* (CONC); Dto. Illapel, Quillaicillo, 18 Sep 1952, *M. Ricardi 2019* (CONC); Altos Mincha-Illapel (Coquimvo), 17 Oct 1965, *J. Lazcano s.n.* (CONC); Dpto. Illapel, Huentelauquén, 31°35'S, 71°32'W, 75 m a.s.l., 20 Oct 1955, *C. Jiles 2812* (CONC); Huentelauquén, quebrada, 31°35'S, 71°32'W, 1 Oct 1957, *G. Monsalve 17* (SGO); Ruta 5N, N caleta Chigualoco Km 246, 31°44'15"S, 71°31'3"W, 113 m a.s.l., 9 Oct 2014, *A. Moreira 2272* (AMM); Dpto. Illapel. Carretera Panamericana, 14 km al norte de Los Vilos. Quebrada El Pangue, 16 Oct 1971, *C. Marticorena*, *R. Rodríguez & E. Weldt 1392* (CONC); 7.3 km on Panamericana N of Turnoff from Panamericana to Los Vilos, between the road and the coast on top of the coastal rocks, 0–40 m a.s.l., 11 Nov 1991, *U. Eggli & B. Leuenberger 1673* (SGO); Más al Norte Quebrada Las Palmas, 18 Sep 1980, *M. Muñoz 1659* (SGO); Quebrada Las Palmas, 22 Sep 1961, *F. Behn s.n.* (CONC); Prov. Aconcagua, Las Palmas, Dec 1976, *R. Palma s.n.* (CONC); Al llegar a la Quebrada de Las Palmas, donde se ven tres palmeras al lado izquierdo del puente, 21 Sep 1980, *M. Muñoz 1686* (SGO); About 10 km north of town [Los Vilos], 31°55'S, 71°30'W, 20 m a.s.l., 3 Oct 1995, *M.F. Gardner & S. Knees 5905* (E); Los Vilos, ca. 20 m a.s.l., 8 Oct 1965, *G. Montero s.n.* (CONC); Los Vilos, ca. 20 m a.s.l., 8 Oct 1965, *G. Montero 7199* (IND); Los Vilos, Sep 1952, *L. Peña s.n.* (CONC); Los Vilos, 30 Oct 1976, *O. Zoellner 9346* (CONC); Los Vilos, 27 Oct 1991, *A. Brinck s.n.* (SGO); Los Vilos, quebrada, 31°55'S, 71°32'W, Oct 1957, *G. Monsalve 72* (SGO); Carretera panamericana, entre Pichidangui y Los Vilos, 12 Oct 1963, *C. Marticorena & O. Matthei 78* (CONC); Carretera Panamericana, 1 km al sur de paso superior Palo Colorado, 15 Oct 1971, *C. Marticorena*, *R. Rodríguez & E. Weldt 1352* (CONC, F); Norte de Pichidangui, 32°5'21.1"S, 71°30'31.4"W, 54 m a.s.l., 6 Oct 2008, *M. Muñoz 5019* (SGO); Peaje al norte de Pichidangui, 32°5'42.6"S, 71°30'19.2"W, 79 m a.s.l., 12 Sep 2008, *M. Muñoz 5001* (SGO); 23.6 km south of Los Vilos on the Panamerican Hwy., 85 m a.s.l., 1 Nov 1990, *T.G. Lammers*, *C.M. Baeza & P. Peñailillo 7678* (CONC); **Valparaíso: [Petorca Province**] Al Norte de Los Molles, 19 Oct 1984, *M. Muñoz 1882* (SGO); **Unknown**: Chile, Jul–Aug 1856, *W.H. Harvey 3898* (E); Chili, Jul 1912, *Unknown s.n.* (L); Without locality, *Unknown 597* (US).

### Names (designations) not validly published

Designations are listed in alphabetic order.

*Schizanthus cruikshanksii* Kunze ex sched., Syn. Pl. Amer austr. Msc., nomen nudum = S. litoralis Phil. var. litoralis. Name only seen on the labels of three specimens collected by Poeppig (In arenos. ad ostia “Rio Aconcagua”, Sep, *E. Poeppig 9* (F! [V0126168F], HAL! [HAL0135743], P! [P00477601])).

*Schizanthus robustus* Phil. ex sched., nomem nudum = *S. alpestris* Poepp. Name only seen on the label of herbarium specimens (Coquimbo, *Unknown s.n.* (B! [destroyed, photo at F! [FOB003062]], K! [000585347, photo at IND! [IND-0107169]], US! [02848562])). All these specimens are samples sent by R.A. Philippi to foreign herbaria.

## Supplementary Material

XML Treatment for Schizanthus

XML Treatment for Schizanthus parvulus

XML Treatment for Schizanthus lacteus

XML Treatment for Schizanthus candidus

XML Treatment for Schizanthus integrifolius

XML Treatment for Schizanthus alpestris

XML Treatment for Schizanthus laetus

XML Treatment for Schizanthus pinnatus

XML Treatment for Schizanthus hookeri

XML Treatment for Schizanthus grahamii

XML Treatment for Schizanthus coccineus

XML Treatment for Schizanthus litoralis var. litoralis

XML Treatment for Schizanthus litoralis var. humilis

XML Treatment for Schizanthus splendens

XML Treatment for Schizanthus porrigens subsp. porrigens

XML Treatment for Schizanthus porrigens subsp. borealis

XML Treatment for Schizanthus carlomunozii

XML Treatment for Schizanthus carlomunozii var. carlomunozii

XML Treatment for Schizanthus carlomunozii var. dilutimaculatus

## Appendix I

**List of classic iconographies**

In the following text we list the publications that contain classic figures of the plants treated in this work. The publications are organised by year under each accepted species.

*Schizanthus candidus* Lindl.

Edwards’s Bot. Reg. 29: tab. 45 (1843).

*Schizanthus grahamii* Gillies ex Hook.

Bot. Mag. 58: tab. 3044 (1831).

Bot. Mag. 58: tab. 3045 (1831) [as *S. retusus*].

Edwards’s Bot. Reg. 18: tab. 1544 (1833) [as *S. retusus*].

Paxton’s Mag. Bot. 1: 5, Plate before page 5 (1834) [as *S. retusus*].

Brit. fl. gard. ser. 2, 3: tab. 201 (1835) [as *S. retusus*].

Bot. Gard. 6: No. 521, T. 131 (1836) [as *S. retusus*].

Rev. Hort. [Paris]. ser. 2, 2: 529, Plate before page 529 (1844).

Rev. Hort. [Paris]. ser. 3, 5: 321, fig. 17 (1851) [as *S. retusus*].

Fl. Serres Jard. Eur. 7: 189, Pl. 712 (1852) [as *S. grahamii* var. *flore albo*].

Gartenflora 12: Taf. 385, fig. 2 y 3 (1863).

Favourite fl. 3: 423, Pl. 203B (1897) [as *S. retusus*].

*Schizanthus hookeri* Gillies ex Graham

Bot. Mag. 58: tab. 3070 (1831).

Schizanthus litoralis Phil. var. litoralis

Exot. Fl. 1: tab. 73 (1823) [as *S. pinnatus*].

Bot. Mag. 50: tab. 2404 (1823) [as *S. pinnatus*].

Brit. fl. gard. 1: tab. 63 (1823–1825) [as *S. pinnatus*].

Schizanthus litoralis Phil. var. humilis (Lindl.) V.Morales & Muñoz-Schick

Brit. fl. gard. ser. 2, 2: tab. 197 (1833) [as S. pinnatus var. humilis].

Edwards’s Bot. Reg. 18: tab. 1562 (1833) [as S. pinnatus var. humilis].

Paxton’s Mag. Bot. 2: 198, Plate before page 198 (1836) [as S. pinnatus var. humilis].

*Schizanthus pinnatus* Ruiz & Pav.

Fl. Peruv. [Ruiz & Pavon] 1: 13, lám. 17 (1798).

Favourite fl. 3: 422, Pl. 203A (1897).

Schizanthus porrigens Graham ex Hook. subsp. porrigens

Exot. Fl. 2(7): tab. 86 (1824).

Fl. Conspic.: tab 32 (1826).

Bot. Gard. 2: No. 126, T. 32 (1828).

Hist. Nat. Vég. (Spach) Atlas: 31, Pl. 77 (1847).

Schizanthus porrigens subsp. porrigens mixed with Schizanthus litoralis var. litoralis (see note below)

Bot. Mag. 51: tab. 2521 (1824).

Bot. Reg. 9: tab. 725 (1824) [as *S. pinnatus*].

Brit. fl. gard. 1: tab. 76 (1823–1825).

**Note.** These illustrations show mixed characters associated with the two species. On one hand, they show the lower lip of the flowers with a darker colour than the upper lip of the corolla (*S. litoralis*) and small spots on the upper lip (middle and lateral lobes) (*S. porrigens*). We think the first illustration can be mixing the characters of S. litoralis var. litoralis and *S. porrigens*, as they were growing together in different gardens during the introduction in the United Kingdom (Hooker 1824, Sweet 1823–1825). On the other hand, the illustrations at the “Botanical Register” and “The British flower garden” may be exaggerating the colour of the lower lip, as sometimes *S. porrigens* exhibits a larger portion of pink between the upper and lower lip.

## Appendix II

**Table:** Distributional ranges and presence of taxa for administrative units in Chile and Argentina.

Administrative units	Altitudinal range (a.s.l.)	Latitudinal range (lat.S)	Chile													Argentina	
Taxa			Tarapacá	Antofagasta	Atacama	Coquimbo	Valparaíso	Metropolitana	O’Higgins	Maule	Ñuble	Biobío	Araucanía	Los Ríos	Los Lagos	Mendoza	Neuquén
*S. alpestris*	180–2800 m	28°50’–32°50’			X	X	X										
*S. candidus*	20–200 m	27°50’–29°00’			X												
S. carlomunozii var. carlomunozii	20–350 m	29°35’–31°10’				X											
S. carlomunozii var. dilutimaculatus	20–350 m	29°25’–32°20’				X	X										
*S. coccineus*	2000–2900 m	33°15’–33°30’						X									
*S. grahamii*	1200–2900 m	33°25’–39°20’						X	X	X	X	X				X	X
*S. hookeri*	900–3200 m	30°35’–37°20’				X	X	X	X	X	X	X				X	X
*S. integrifolius*	800–2800 m	26°20’–30°10’			X	X											
*S. lacteus*	20–900 m	23°30’–26°00’		X													
*S. laetus*	20–900 m	20°40’–26°00’	X	X													
S. litoralis var. litoralis	10–100 m	32°30’–33°10’					X										
S. litoralis var. humilis	0–20 m	32°20’–32°35’					X										
*S. parvulus*	100–900 m	31°20’–32°10’				X											
*S. pinnatus*	30–2000 m	33°35’–40°30’					X	X	X	X	X	X	X	X	X		
S. porrigens subsp. porrigens	20–1800 m	29°25’–29°50’				X	X	X	X								
S. porrigens subsp. borealis	170–600 m	32°05’–34°20’				X											
*S. splendens*	350–1800 m	30°10’–31°00’				X											
